# Upscaling X-ray nanoimaging to macroscopic specimens

**DOI:** 10.1107/S1600576721000194

**Published:** 2021-02-19

**Authors:** Ming Du, Zichao (Wendy) Di, Doǧa Gürsoy, R. Patrick Xian, Yevgenia Kozorovitskiy, Chris Jacobsen

**Affiliations:** a Advanced Photon Source, Argonne National Laboratory, Argonne, IL 60439, USA; b Mathematics and Computer Science Division, Argonne National Laboratory, Argonne, IL 60439, USA; c Department of Electrical Engineering and Computer Science, Northwestern University, Evanston, IL 60208, USA; d Department of Neurobiology, Northwestern University, Evanston, IL 60208, USA; e Chemistry of Life Processes Institute, Northwestern University, Evanston, IL 60208, USA; f Department of Physics and Astronomy, Northwestern University, Evanston, IL 60208, USA

**Keywords:** X-ray microscopy, phase contrast X-ray imaging

## Abstract

Upscaling X-ray nanoimaging to macroscopic specimens has the potential for providing insights across multiple length scales, but its feasibility has long been an open question. By combining the imaging requirements and existing proof-of-principle examples in large-specimen preparation, data acquisition and reconstruction algorithms, the authors provide imaging time estimates for howX-ray nanoimaging can be scaled to macroscopic specimens. To arrive at this estimate, a phase contrast imaging model that includes plural scattering effects is used to calculate the required exposure and corresponding radiation dose. The coherent X-ray flux anticipated from upcoming diffraction-limited light sources is then considered. This imaging time estimation is in particular applied to the case of the connectomes of whole mouse brains. To image the connectome of the whole mouse brain, electron microscopy connectomics might require years, whereas optimized X-ray microscopy connectomics could reduce this to one week. Furthermore, this analysis points to challenges that need to be overcome (such as increased X-ray detector frame rate) and opportunities that advances in artificial-intelligence-based ‘smart’ scanning might provide. While the technical advances required are daunting, it is shown that X-ray microscopy is indeed potentially applicable to nanoimaging of millimetre- or even centimetre-size specimens.

## Abstract 2

In the era of diffraction-limited storage rings, can X-ray nanoimaging be extended to millimetre- or even centimetre-sized specimens such as whole mouse brains? The authors believe so and provide size-dependent imaging time and resource estimates based on calculated flux requirements and recent method developments in related disciplines.

## 1. Introduction   

One naturally thinks of microscopy as applying to small objects. We ask here a different question: how large an object can one realistically image using X-ray microscopy at synchrotron light sources?

### 1.1. A specific example: X-ray microscopy connectomics   

While this is a question of interest to studies of a wide variety of materials, we will use one particular challenge as a touchstone for our considerations: can we determine the complete ‘wiring diagram,’ or connectome, of a whole mouse brain using X-ray microscopy? Our understanding of brain function relies on a detailed map of brain structure and connectivity at various length scales. This map is currently unevenly sampled and incomplete, especially for large vertebrate brains (Morgan & Lichtman, 2013 ▸; DeWeerdt, 2019 ▸). The information one can gain is also relevant for designing learning and neuromorphic computing architectures that harness the engineering efficiency and individual ‘component’ failure tolerance of nature (Helmstaedter, 2015 ▸; Hassabis *et al.*, 2017 ▸; Abbott *et al.*, 2020 ▸). Although the connectome can be considered on different spatial scales owing to the hierarchical organization of the brain (Zeng, 2018 ▸), we refer here to the connectome with synaptic resolution. That is because one needs to see the synapses themselves to be sure of the ‘wiring diagram’ (Kasthuri *et al.*, 2015 ▸). Humans are estimated to have about 8.6 × 10^10^ neurons in the entire central nervous system, about 2 × 10^10^ in the neocortex, and perhaps 6 × 10^14^ synapses in the entire central nervous system (Silbereis *et al.*, 2016 ▸), presenting a currently insurmountable connectomics problem. The field has therefore focused efforts on reconstructing segments of smaller vertebrate connectomes, with a particular emphasis on the mouse brain since mice are the most common vertebrate model organism in biomedical research. There are about 7.2 × 10^8^ synapses per mm^3^ in mouse cortex, or about 8.1 × 10^10^ total synapses in a volume of 112 mm^3^ (Schüz & Palm, 1989 ▸). The typical volume of a synapse in mouse is about 1.2 × 10^5^ nm^3^ (Kasthuri *et al.*, 2015 ▸), corresponding to a diameter of 77 nm for a perfect half-sphere (which a synapse is not). Therefore synapses take up a fractional volume of about 9 × 10^−5^ in the mouse cortex, and their collective spatial distribution (without considering subtypes) has been shown to be close to random in rats (Anton-Sanchez *et al.*, 2014 ▸).

Unambiguous identification of dense synaptic organizations in millimetre-thick specimens of vertebrate brain is beyond the capability of light microscopy [though tissue clearing methods (Richardson & Lichtman, 2015 ▸; Ueda *et al.*, 2020 ▸) can help extend this considerably]. The fundamental property that limits thick specimen imaging using visible light is the transport mean free path (determined in part by the 1/*e* distance for plural scattering; Helmchen & Denk, 2005 ▸), which is 50–100 µm at λ = 630 nm for extracted brain tissue (Taddeucci *et al.*, 1996 ▸; Yaroslavsky *et al.*, 2002 ▸) and 200 µm at λ = 800 nm *in vivo* (Oheim *et al.*, 2001 ▸).

Owing to its capability for much higher spatial resolution, and its commercial availability, the dominant technique for mapping connectomes of different species has been electron microscopy (EM). However, in electron microscopy the thickness limit is set by the mean free path for inelastic scattering, which is about 0.2 µm in ice at 120 keV (Angert *et al.*, 1996 ▸; Grimm *et al.*, 1996 ▸) and similar distances in plastic. Plural scattering then dominates by the time thicknesses of 1 µm are reached (Langmore & Smith, 1992 ▸). As a result, large-volume imaging has to be carried out using either serial sections or serially exposed faces of a volume (Kornfeld & Denk, 2018 ▸). Several studies have imaged roughly (0.2 mm)^3^ subregions of mouse brain (Lichtman & Denk, 2011 ▸; Mikula & Denk, 2015 ▸; Kasthuri *et al.*, 2015 ▸; Mikula, 2016 ▸; Motta *et al.*, 2019 ▸). However, upscaling these results to whole mouse brain imaging is challenging; one estimate for diamond-cut blockface scanning electron microscopy is that it would take eight years to image a volume of (1 mm)^3^ at 16 nm voxel resolution (Xu *et al.*, 2017 ▸), while another estimate is that it would take 12 years for the same volume at 10 × 10 × 25 nm resolution (Titze & Genoud, 2016 ▸). The time for imaging (1 mm)^3^ could conceivably drop to less than one year using multi-beam scanning electron microscopy (Eberle & Zeidler, 2018 ▸), and recently a highly automated pipeline has been used to image 1 mm^3^ in less than six months using six transmission electron microscopes (Yin *et al.*, 2020 ▸). However, serial sectioning is still accompanied by inherently anisotropic resolution (Kreshuk *et al.*, 2011 ▸; Kornfeld & Denk, 2018 ▸) and unavoidable knife-cutting artifacts (Khalilian-Gourtani *et al.*, 2019 ▸), which can complicate faithful 3D characterization of the connectome.

X-ray microscopy offers a potential alternative for connectomics studies. The attenuation length of 15 keV X-rays in soft tissue is 0.65 cm (Henke *et al.*, 1993 ▸), and phase contrast dominates over inelastic scattering for thicknesses in the centimetre range (Du & Jacobsen, 2018 ▸, 2020 ▸). In light materials like tissue and plastic at photon energies below about 15 keV, the cross section for photoelectric absorption is larger than that for elastic and inelastic scattering (Hubbell *et al.*, 1975 ▸), so that images are largely free of the ‘blur’ caused by plural scattering (Du & Jacobsen, 2018 ▸; Jacobsen, 2020 ▸). As a result, near-micrometre-scale resolution X-ray tomography has already been utilized for several neuroanatomy studies of significant portions of, or even whole, mouse brains (Mizutani *et al.*, 2016 ▸; Dyer *et al.*, 2017 ▸; Töpperwien *et al.*, 2017 ▸; Fonseca *et al.*, 2018 ▸; Masís *et al.*, 2018 ▸; Depannemaecker *et al.*, 2019 ▸; Massimi *et al.*, 2019 ▸), as well as on 43 mm^3^ sub-volumes of human brain (Hieber *et al.*, 2016 ▸). Several volume-stitching schemes allow this to be extended to 1 µm resolution on whole mouse brains, yielding petavoxel volume reconstructions (Vescovi *et al.*, 2018 ▸; Du *et al.*, 2018 ▸; Miettinen *et al.*, 2019 ▸), while sub-micrometre resolution has been demonstrated on smaller brain tissue specimens (Yang *et al.*, 2018 ▸; Khimchenko *et al.*, 2018 ▸; Kuan *et al.*, 2020 ▸), including specimens with fixation but no staining (Shahmoradian *et al.*, 2017 ▸). (The question of staining in connectomics is addressed in Section 6.1 below.) At the level of single algae cells imaged in a frozen hydrated state at liquid nitrogen temperature, 18 nm resolution has been achieved in 2D transmission images (Deng, Vine *et al.*, 2017 ▸), and 45 × 45 × 55 nm resolution in three dimensions (Deng *et al.*, 2018 ▸).

### 1.2. Materials science example   

For more radiation-hard specimens, 18 nm resolution has been obtained when imaging copper features through 300 µm of silicon (Deng, Hong *et al.*, 2017 ▸), and 8 nm resolution through 130 µm silicon (Deng *et al.*, 2019 ▸), while 15 nm isotropic resolution has been obtained in 3D images of extracted subregions of integrated circuits (Holler *et al.*, 2017 ▸).

### 1.3. The question at hand   

Connectomics of whole vertebrate brains provides one example challenge where one would like to upscale nanoscaleX-ray imaging to accommodate macro-sized objects. Another example involves whole integrated circuits, where one might want to verify that they have been manufactured as designed, rather than having ‘Trojan horse’ circuitry nefariously inserted (Adee, 2008 ▸; Xiao *et al.*, 2016 ▸). Recent X-ray microscopy studies of the failure mechanisms of battery materials (Weker *et al.*, 2017 ▸; Yu *et al.*, 2018 ▸) have usually involved studies of single particles; by extending the field of view, one can go from microscopic examples to whole-battery-cell statistics. Is it realistic to extend X-ray nanoscale imaging up to millimetre- or even centimetre-sized objects within reasonable imaging times? This is the question we address below.

## 2. Fluence and radiation dose considerations   

The first requirement for transmission imaging of increasingly thick specimens is to have sufficient image contrast and acceptable radiation dose. With thicker specimens, one must use multi-keV X rays to allow for penetration of the beam. At these photon energies, phase contrast provides the most favorable imaging mechanism (Schmahl & Rudolph, 1987 ▸; Davis *et al.*, 1995 ▸; Du & Jacobsen, 2018 ▸, 2020 ▸).

### 2.1. Estimating the required exposure   

**Figure 1:**
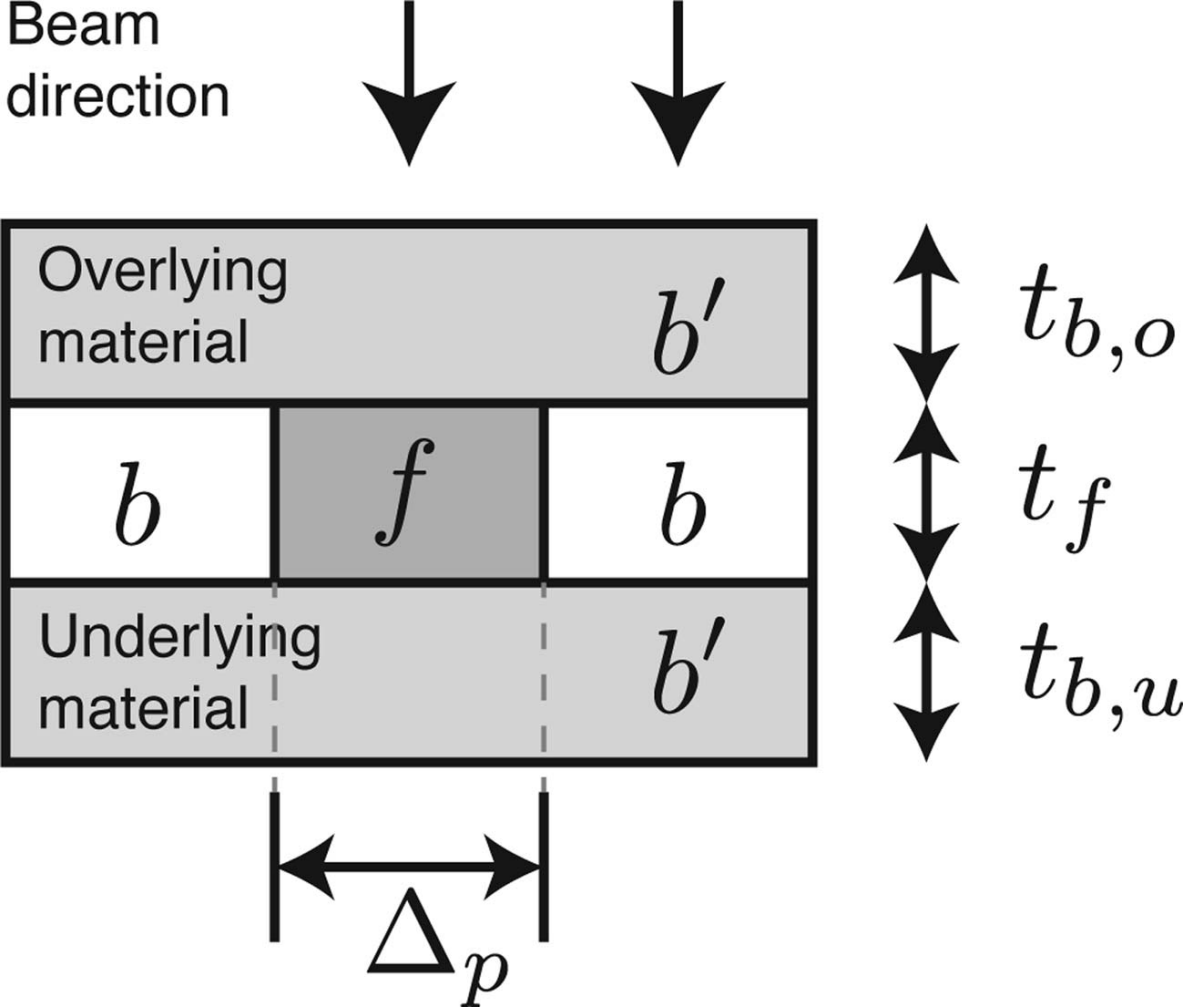
Schematic of the specimen used for our calculations. Within the ‘feature slab’ of thickness *t*_*f*_, we assume that we have a pixel of width Δ_p_ with a feature *f* next to pure background material *b*. In the planes above and below, we may have a mixed background material *b*′. In the case of copper features *f* in a matrix of silicon, we assume that both *b* and *b*′ are silicon. In the case of a biological specimen, we assume that the feature *f* is protein embedded in a background *b* of ice, while the mixed background material *b*′ above and below (referred to as ‘tissue’ in this manuscript) is 70% ice and 30% protein in accordance with the typical water fraction of the human brain (Shah *et al.*, 2008 ▸).

For thin-specimen imaging, several investigators have provided estimates for the required exposure for a variety of X-ray microscopy methods (Sayre *et al.*, 1976 ▸; Shen *et al.*, 2004 ▸; Howells *et al.*, 2009 ▸; Schropp & Schroer, 2010 ▸; Villanueva-Perez *et al.*, 2016 ▸). These calculations make use of literature values (Henke *et al.*, 1993 ▸; Schoonjans *et al.*, 2011 ▸) for the X-ray refractive index
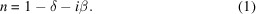
Following earlier work (Du & Jacobsen, 2018 ▸, 2020 ▸), we use a simple model for Zernike phase contrast of a specimen as shown in Fig. 1 ▸; this also provides a good approximation for various forms of coherent diffraction imaging [see for example Section 4.8.5 of Jacobsen (2020 ▸)]. That is, we assume that a feature material *f* is within a background material *b* in a layer of thickness *t*_*f*_, with a pixel size of Δ_p_. Over and under this plane of interest in a tomographic reconstruction, we assume that there is a thickness 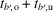 of a mixed background material *b*′. A simple estimate [equation 39 of Du & Jacobsen (2018 ▸, 2020 ▸), or equation 4.267 of Jacobsen (2020 ▸)] of the number of photons required for phase contrast imaging of a feature of thickness *t*_*f*_ in a thickness *b*′ of mixed background material is 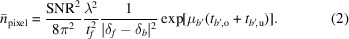
The signal-to-noise ratio is assumed to be 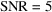, following the Rose (1946 ▸) criterion and the choice of many previous studies. The X-ray linear absorption coefficient is given by μ = 4πβ/λ, where λ = *hc*/*E* is the X-ray wavelength corresponding to the photon energy *E*, and *hc* = 1239 eV nm is Planck’s constant times the speed of light. The radiation dose *D*_*f*_ imparted to the feature by this exposure [equation 92 of Du & Jacobsen (2018 ▸, 2020 ▸)] is given by 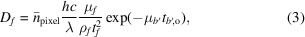
where ρ_*f*_ is the density of the feature material. The radiation dose *D*_*f*_ is usually expressed in Gray, where 1 Gy corresponds to 1 J of ionizing energy absorbed per kilogram of material.

**Figure 2:**
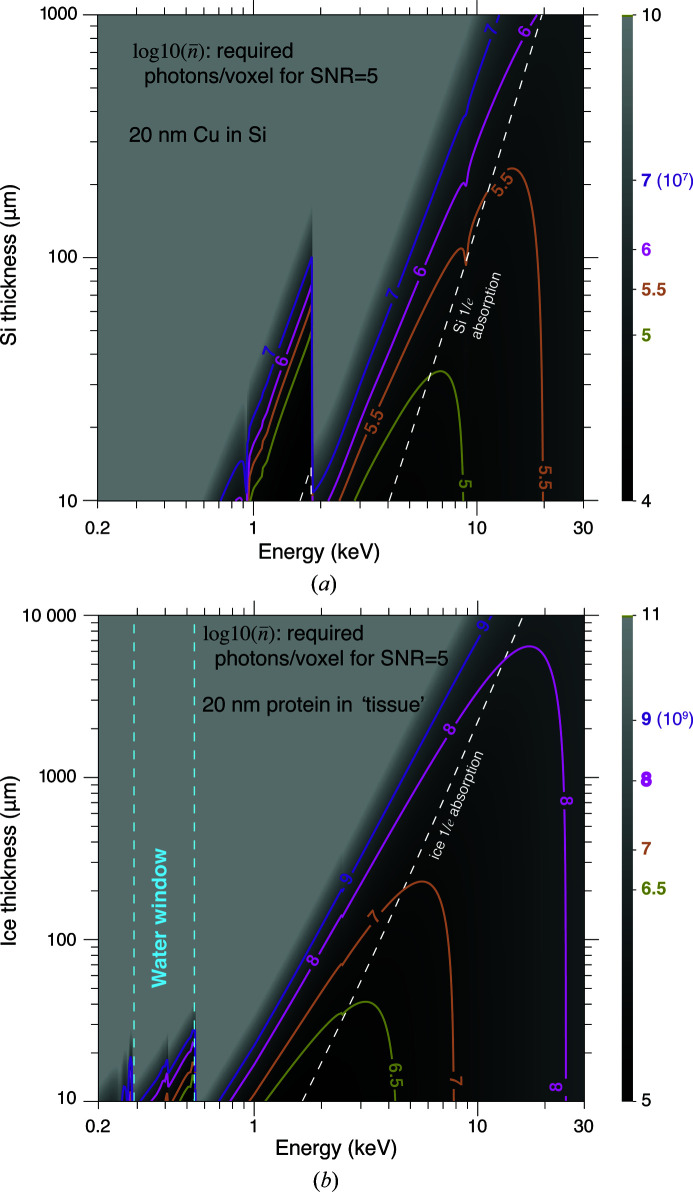
Calculations for the required number of incident photons per pixel 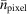 for SNR = 5 imaging at δ_r_ = 20 nm spatial resolution. These calculations are for 2D imaging of copper features in silicon (*a*) to represent an integrated circuit and for imaging protein features adjacent to ice with over- and underlying layers (Fig. 1 ▸) of 70% water/30% ice as tissue (*b*) to represent a biological specimen. These figures show contour lines for 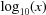, so that *x* = 7 refers to a contour of 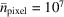; the underlaid grayscale image also displays *x*. Also shown as a white dashed line is the 1/*e* attenuation length μ^−1^(*E*) of the background material (either silicon or tissue) as a function of photon energy [equation (4)]. (*a*) shows the effect of the Si *K* absorption edge at 1.84 keV, while (*b*) shows the ‘water window’ between the carbon (0.29 keV) and oxygen (0.54 keV) *K* absorption edges.

**Figure 3:**
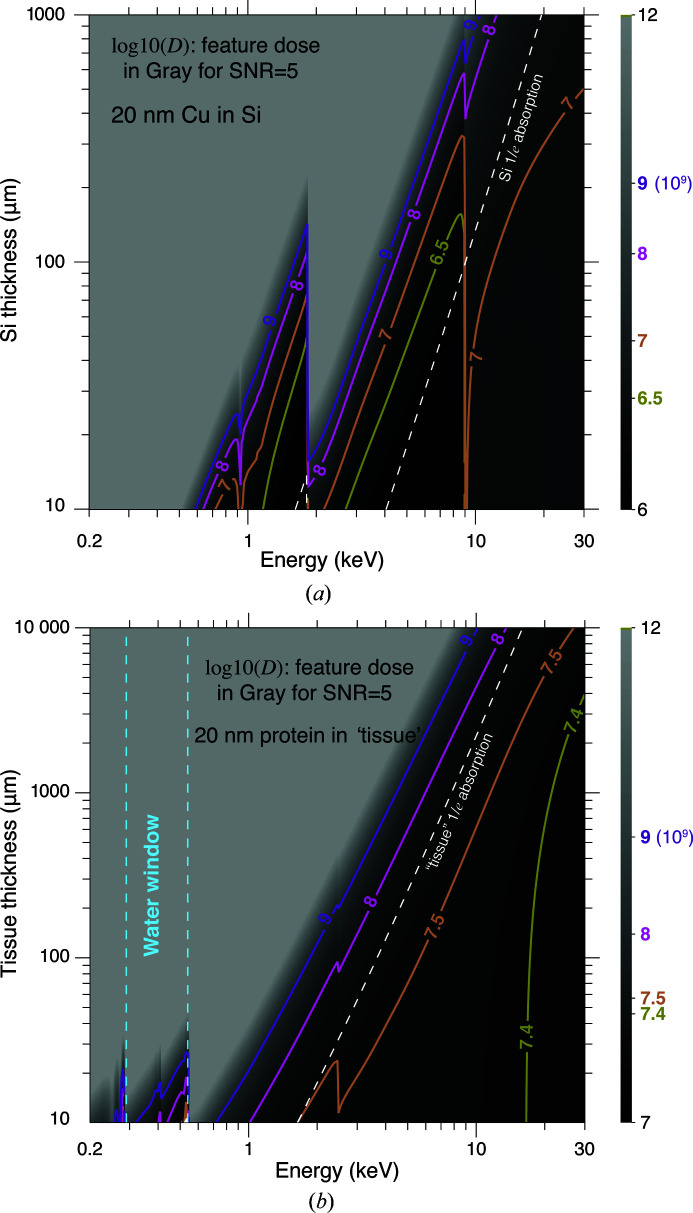
Calculations for the radiation dose *D*_*f*_ to the feature, using the required number of incident photons per pixel 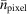 for δ_r_ = 20 nm spatial resolution imaging, as shown in Fig. 2 ▸. These calculations are for 2D imaging of copper features in silicon (*a*) to represent an integrated circuit and for imaging protein features adjacent to ice with over- and underlying layers (Fig. 1 ▸) of 70% water/30% ice as tissue (*b*) to represent a biological specimen. These figures show contour lines for 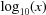, so that *x* = 7 refers to a contour of *D* = 10^7^; the underlaid grayscale image also displays *x*. Also shown as a white line is the 1/*e* attenuation length μ^−1^(*E*) of the background material (either silicon or tissue) as a function of photon energy [equation (4)].

For thicker specimens, a more complete treatment of the per-pixel illumination 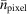 and associated radiation dose *D*_*f*_ to the feature must account for plural elastic scattering as well as inelastic scattering. It must also include absorption contrast, which is sometimes more favorable at lower photon energies. Using this more complete calculation [equations 86–89 of Du & Jacobsen (2020 ▸)], we show in Fig. 2 ▸ the required number of photons per pixel, 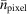, and in Fig. 3 ▸ the radiation dose to the feature, *D*_*f*_, for two examples of X-ray nanoscale imaging of macroscale objects:

(i) The first example is of imaging copper features in an integrated circuit, where the circuitry is usually confined to a very small plane in the entire chip, so we will assume that the feature *f* is pure copper in a background material *b* of silicon. The mixed background material *b*′ is also mainly silicon in this case.

(ii) The second example of imaging a biological specimen is somewhat different. We may have a dense organelle with mainly water on either side, so we will assume that the feature *f* has the stoichiometric composition of a representative protein formed from the average of all 20 amino acids. This leads to a composition of H_48.6_C_32.9_N_8.9_O_8.9_S_0.6_ with a density when dehydrated of 1.35 g cm^−2^ (London *et al.*, 1989 ▸). The background *b* is assumed to be of amorphous ice with a density of 0.92 g cm^−3^ for frozen hydrated biological specimens (Dubochet *et al.*, 1982 ▸) (we assume that some new form of high-pressure freezing can be used to prepare thicker specimens than are now typical in cryogenic imaging). In the planes above and below, we assume that we have ‘tissue’ as a background material *b*′ with a composition of 70% ice and 30% protein, since brain tissue is about 70% water (Shah *et al.*, 2008 ▸) while single cells tend to be about 75% water (Fulton, 1982 ▸; Luby-Phelps, 2000 ▸).

We refer to these two examples as ‘Cu in Si’ and ‘protein in tissue’ in subsequent sections.

**Figure 4:**
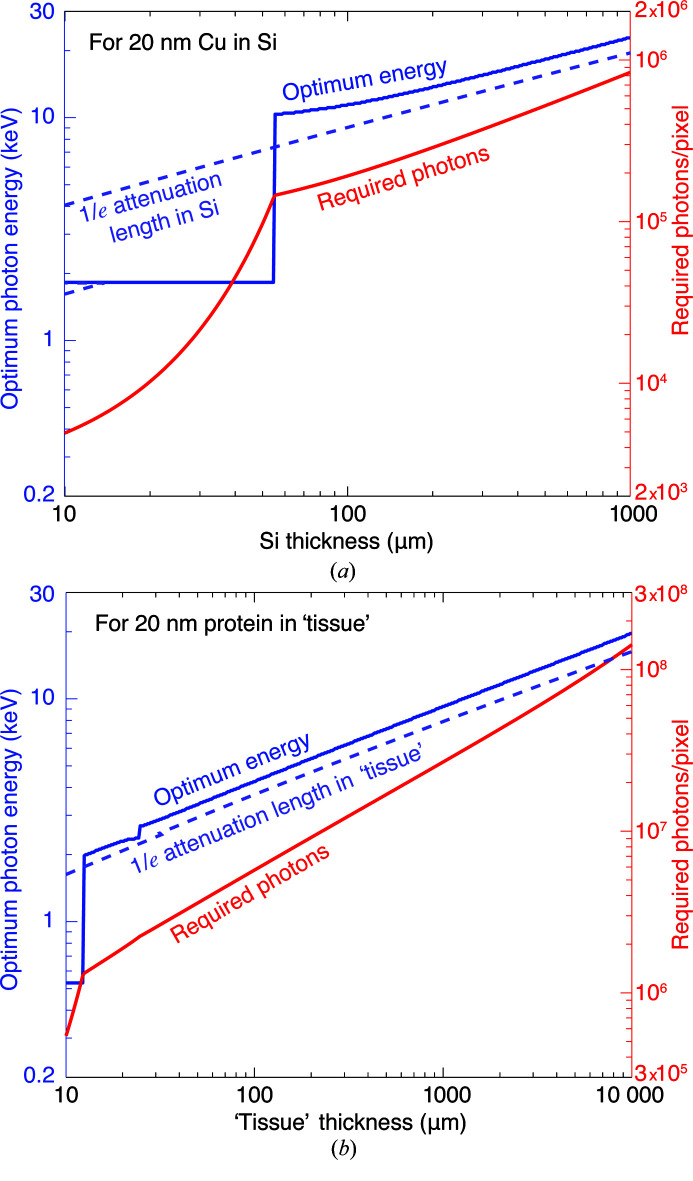
Optimum photon energy *E*_*n*_ which minimizes the required number of incident photons per pixel 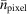 (left axis) and the corresponding value of 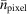 (right axis) for imaging copper in silicon (*a*) and protein in tissue (*b*). These values were obtained from the calculations of 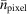 shown in Fig. 2 ▸ as a function of both background material thickness and photon energy, assuming SNR = 5 and δ_r_ = 20 nm. Also shown is the energy *E*_est_ found by setting μ^−1^(*E*_est_) of equation (4) equal to the total thickness *t* of the background material. As discussed in Section 3.1, the required illumination per pixel in 2D imaging shown here is approximately the same as the integrated illumination per voxel in 3D imaging.

As seen in Fig. 2 ▸, once one knows the overall thickness 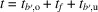 of the specimen that the X-ray beam must penetrate, the optimum photon energy can be estimated by matching *t* to the energy-dependent X-ray attenuation length 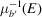 of the background material, since the Lambert–Beer law 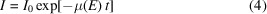
describes X-ray absorption. While the specimen becomes too absorptive at lower photon energies for optimum imaging, at higher photon energies the contrast begins to be reduced (thus leading to a requirement for a larger number of incident photons per pixel 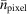), and furthermore the coherent flux is reduced at higher energies, as will be discussed in Section 4. Figs. 2 ▸ and 3 ▸ show a dashed line plot of the photon energy *E*_est_ for which 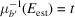 for the background material, demonstrating that this condition provides a reasonably good estimate of the photon energy that requires the fewest photons for imaging. A more exact result for each overall sample thickness *t* is obtained by choosing the minimum fluence 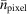 from Fig. 2 ▸, and also noting the photon energy *E*_*n*_ at which this minimum is obtained. These more exact results for 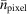 and *E*_*n*_ are shown in Fig. 4 ▸, along with *E*_est_.

The radiation dose shown in Fig. 3 ▸ is that imparted to the feature material. For one viewing angle, the incident fluence will be higher on the surface of the background material facing into the illumination and lower at the exit surface owing to attenuation of the beam. However, when the specimen is rotated relative to the illumination direction as is required for tomography, this dose imbalance will even out to some degree. Furthermore, since the conditions for optimum imaging are well approximated by having μ^−1^(*E*) = *t*, the angle-integrated dose to the background material near the center is also similar to the average surface dose.

The X-ray transmission-based methods considered above, like absorption and phase contrast imaging, are not the only options for thick-specimen studies. X-ray fluorescence offers the opportunity to image specific elemental concentrations in a specimen (Sparks, 1980 ▸; Jacobsen, 2020 ▸), and there are proposals to develop X-ray Compton microscopy for reduced-dose imaging using inelastic scattering (Villanueva-Perez *et al.*, 2018 ▸). However, these other imaging modes still require that some fraction of the illumination beam penetrate through the specimen in order to illuminate at least the mid-point (and preferably the downstream surface) in a tomography experiment, so one will make choices of the incident beam energy similar to those shown for Zernike phase contrast in Fig. 2 ▸.

### 2.2. Comparison with experimental results   

The above estimates are quite consistent both with simulation studies (Du, Gürsoy & Jacobsen, 2020 ▸) and with experimental results. In 2D X-ray ptychography experiments (Deng, Vine *et al.*, 2017 ▸) with frozen hydrated algae at 5 keV, a calculation based on the above methodology, using literature X-ray refractive index values for protein and ice and a signal:noise ratio of 5:1, gave an estimate for a required exposure of 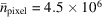
 photons for δ_r_ = 20 nm, whereas the experimental exposure for δ_r_ = 18 nm resolution was 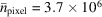. Similarly, 2D imaging of δ_r_ = 20 nm Cu features in 240 µm-thick Si yielded an estimate of 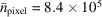, whereas an experimental exposure of 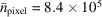
 photons per (20 nm)^2^ yielded an achieved resolution of δ_r_ = 18 nm (Deng, Hong *et al.*, 2017 ▸).

While one may be concerned that experimental complications (such as illumination fluctuation, partial coherence and sample stage position errors) may undermine the accuracy of our dose estimation, computational methods can compensate for these imperfections (Guizar-Sicairos & Fienup, 2008 ▸; Maiden, Humphry, Sarahan *et al.*, 2012 ▸; Zhang *et al.*, 2013 ▸; Pelz *et al.*, 2014 ▸; Deng, Nashed *et al.*, 2015 ▸; Odstrčil *et al.*, 2018 ▸). Furthermore, denoising approaches including Bayesian algorithms (Nikitin *et al.*, 2019 ▸) and deep neural networks (Aslan *et al.*, 2020 ▸) have been shown to be effective against both photon noise and structured noise. Thus one may be able to further relax the requirement on fluence and dose.

### 2.3. Radiation dose limits   

The calculations given above provide a relationship between specimen thickness, spatial resolution, and both the incident number of photons 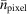 and the radiation dose *D*_*f*_ in Gy. They also assume 100% efficiency of the imaging system. What radiation dose is tolerable? The topic is complex [see for example chapter 11 of Jacobsen (2020 ▸)]. Different polymers show differing dose sensitivity, but the critical dose for mass loss in a relatively sensitive polymer (polymethyl­meth­acryl­ate) is about 6 × 10^8^ Gy at 100 K (Beetz & Jacobsen, 2003 ▸). X-ray diffraction spots from protein crystals studied at liquid nitrogen temperature start to fade out at doses of about 2 × 10^7^ Gy as one begins to affect a significant fraction of the bonds in macromolecules (Henderson, 1990 ▸). However, microscopy at tens of nanometres spatial resolution is limited not by bond breaking but by mass loss or rearrangement at much longer length scales, so that little observable change has been observed in 30 nm-resolution images of frozen hydrated algae at doses of 2 × 10^9^ Gy (Deng, Vine *et al.*, 2015 ▸) or in 100 nm-resolution images of frozen hydrated fibroblasts at doses of up to about 10^10^ Gy (Maser *et al.*, 2000 ▸). Frozen hydrated specimens exhibit a destructive ‘bubbling’ phenomenon at the high dose rate present in electron microscopy (∼10^11^ Gy; Dubochet *et al.*, 1982 ▸; Leapman & Sun, 1995 ▸). In materials science specimens, doses of about 10^9^ Gy are associated with changes in the size of Li-S battery particles (Nelson *et al.*, 2013 ▸), as well as a reduction in Bragg diffraction from silicon-on-insulator materials (Polvino *et al.*, 2008 ▸). Therefore we will assume that the maximum dose that a specimen can tolerate is *D*_max_ = 10^9^ Gy. As can be seen in Fig. 3 ▸, this dose is not exceeded for SNR = 5 imaging at δ_r_ = 20 nm spatial resolution at the photon energies that minimize the number of photons required.

### 2.4. Dose-efficient imaging with ptychography   

Given the limits that radiation dose sets, and the conflicting requirements that high doses are required for high-resolution imaging as discussed in Section 2.1, it is important to use a dose-efficient approach for nanoimaging of thick specimens. While other approaches to produce X-ray phase contrast exist (Mokso *et al.*, 2007 ▸; Holzner *et al.*, 2010 ▸), we identify coherent diffraction imaging as a favorable choice, since it requires no lossy resolution-limiting optics between the specimen and uses an efficient direct-to-silicon pixel array detector. Moreover, the scanned coherent beam approach of ptychography offers robust image reconstruction of phase objects without the requirement of a finite sample extent (Rodenburg *et al.*, 2007 ▸). Therefore, we concentrate in what follows on the use of X-ray ptychography for dose-efficient thick-specimen imaging.

## 3. Three-dimensional imaging considerations   

**Figure 5:**
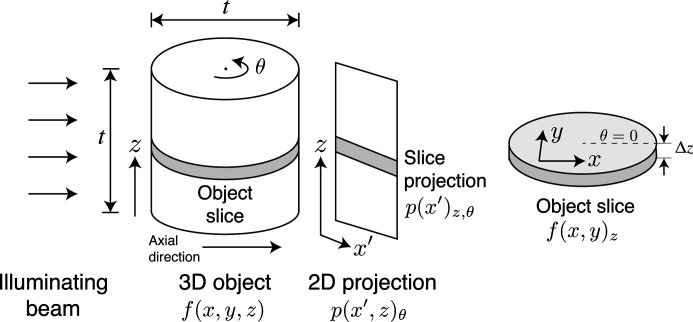
Geometry considered for conventional tomography of a cylindrical object of diameter *t* and height *t* at a synchrotron light source. As the object is rotated about the 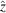 axis, projection images are obtained as one row in the 2D detector collects information about one slice *f*(*x*, *y*)_*z*_ of the object, with a slice thickness of Δ*z*. The collection of projection images provides information in the {*X*, *Y*} plane of the Fourier transform of the object slice. Adapted from Fig. 8.1 of Jacobsen (2020 ▸).

The estimates for the required number of incident photons per pixel 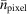 of equation (2) and the corresponding radiation dose *D*_*f*_ were for 2D imaging of features within a uniform thick specimen. In fact, for a truly 3D specimen with features contained throughout, a single 2D projection image will yield a bewildering overlay of features contained throughout the depth of the specimen. Therefore nanoscale imaging of thick specimens will necessarily require the acquisition of a large number *N*_θ_ of projection images with the specimen rotated, typically about an axis orthogonal to the direction of the illuminating beam as shown in Fig. 5 ▸ (although alternative approaches such as laminography have advantages for specimens on thick planar substrates; Helfen *et al.*, 2005 ▸; Xu *et al.*, 2012 ▸; Holler *et al.*, 2019 ▸).

### 3.1. Dose fractionation   

One might normally think that the acquisition of *N*_θ_ projection images will involve illumination with 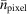 photons per pixel for each image, thus multiplying by *N*_θ_ both the required flux and the radiation dose *D*_*f*_ estimates of Figs. 3 ▸ and 4 ▸. However, this is not the case, because tomographic reconstruction involves a summation into each voxel of the information from all projections. This was realized by Hegerl & Hoppe (1976 ▸) in the case of electron microscopy, who stated (substituting our use of *N*_θ_ for their use of *K* for the number of projections) ‘A three-dimensional reconstruction requires the same integral dose as a conventional two-dimensional micrograph provided that the level of significance and the resolution are identical. The necessary dose *D* for one of the *N*_θ_ projections in a reconstruction series is, therefore, the integral dose divided by *N*_θ_.’

This principle has been stretched further in single-particle electron microscopy (Frank, 1975 ▸; Frank *et al.*, 1988 ▸; Cheng, 2015 ▸), where thousands of individual very noisy 2D images are combined to yield high-resolution 3D structures. Dose fractionation is valid only if one can correctly align individual noisy 2D images onto the 3D reconstruction volume (McEwen *et al.*, 1995 ▸). However, this is routinely done in single-particle microscopy as noted above, and in tomography using methods such as iterative reprojection (Dengler, 1989 ▸; Gürsoy *et al.*, 2017 ▸) and numerical optimization (Di *et al.*, 2019 ▸).

### 3.2. Pixels, voxels and tilts   

We now consider the question of imaging a cylindrical specimen with diameter *t* and height *t* at a spatial resolution of δ_r_ as shown in Fig. 5 ▸. To meet the conditions of Nyquist sampling (Nyquist, 1928 ▸; Shannon, 1949 ▸), the voxel size Δ_v_ should be 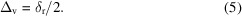
At each angular orientation of the specimen, the imaging field width should meet the condition *N*Δ_v_ = *t*, so we can write the number of pixels *N* across the object per viewing angle as 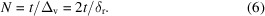
If we set the cylinder height to be the same distance *t*, equation (6) also gives the number of voxels in that direction.

While we will consider beyond-depth-of-focus imaging in Section 5.1 below, let us first consider the case where an image from one viewing angle delivers a pure projection through the object: there is no axial information from that viewing angle. Following the convention of Fig. 5 ▸, we assume that the rotation axis is vertical (the 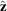 direction) and that the horizontal direction (perpendicular to the beam direction) is the 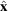 direction. In that case, the *N* × 1 pixels collected in one row in the detector have a Fourier transform with data in *N* × 1 pixels, where the latter dimension corresponds to a spatial frequency of zero in the axial direction. As the specimen is rotated through each angle θ, *N* × 1 pixel contributions are made at that angle to the {*X*, *Y*} Fourier space representation of the object slice *f*(*x* *y*)_*z*_. One can then show that, to completely fill in all voxels out to a radius of *N*/2 from the center zero-spatial-frequency voxel in the 3D Fourier transform, one must record data over a number of projection angles of 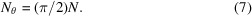
This is known as the Crowther limit (Crowther *et al.*, 1970 ▸). Satisfying the Crowther limit is especially important when using filtered backprojection for rapid tomographic reconstruction. While iterative reconstruction algorithms can incorporate *a priori* information about the object and thus greatly reduce missing-angle artifacts (Kak & Slaney, 1988 ▸), and artificial-intelligence-based methods can be used to fill textures from acquired angles into unacquired angles via inpainting (Kim *et al.*, 2010 ▸; Yoo *et al.*, 2019 ▸; Ding *et al.*, 2019 ▸), the fundamentals of the information contained in projections remains unchanged so that the 3D reconstruction will lose detail or accuracy if equation (7) is not satisfied. Modification of the Crowther criterion for the case of beyond-depth-of-focus imaging will be considered in Section 5.1.

### 3.3. From pixel illumination to total illumination   

How many photons are required to illuminate the entire object? Consider first the case of one object slice as shown in Fig. 5 ▸. As discussed in Section 3.1, the requirement of using 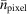 photons to illuminate one pixel can be satisfied by distributing these photons over all *N*_θ_ rotation angles. Thus the required exposure of a voxel in a slice per rotation angle is 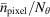, and since each slice projection contains *N* pixels the total number of photons required to illuminate the slice from each angle is given by 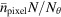. Data collection over all *N*_θ_ angles then gives a net illumination requirement for the object slice of 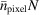. Equal illumination must be provided for all of the *N* object slices in the 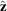 direction, yielding a total illumination requirement of 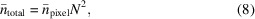
where 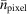 is found from equation (2) and *N* is given by equation (6).

## 4. X-ray source considerations   

As discussed in Section 2.4, X-ray ptychography provides a dose-efficient way to obtain phase contrast images. However, this means one must use high-brightness X-ray sources, since the spatially coherent flux Φ_c_ available from the source is given by its brightness *B* multiplied by the X-ray wavelength λ squared (Green, 1976 ▸; Kondratenko & Skrinsky, 1977 ▸), or 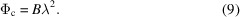
This is because the full-width full-angle phase space area of a spatially coherent illumination mode is given by 1λ in each transverse direction based on a criterion of preserving near-diffraction-limited focusing in a scanning microscope (Jacobsen *et al.*, 1992 ▸; Winn *et al.*, 2000 ▸; Jacobsen, 2020 ▸). Dramatic increases in coherent flux are becoming available with the advent of diffraction-limited storage rings (Eriksson *et al.*, 2014 ▸) where the electron beam emittance is approximately equal to the X-ray wavelength λ in both the horizontal and vertical directions. Even higher time-averaged brightness is available from X-ray free-electron lasers (XFELs), but time averaging hides the fact that they deliver copious numbers of photons in beam pulses lasting tens of femtoseconds (far too short a time to carry heat away) so that each pulse can cause photoablation (David *et al.*, 2011 ▸). This makes XFELs poorly matched to the goal of imaging the same specimen with the beam scanned across many positions at each of many rotation angles.

**Figure 6:**
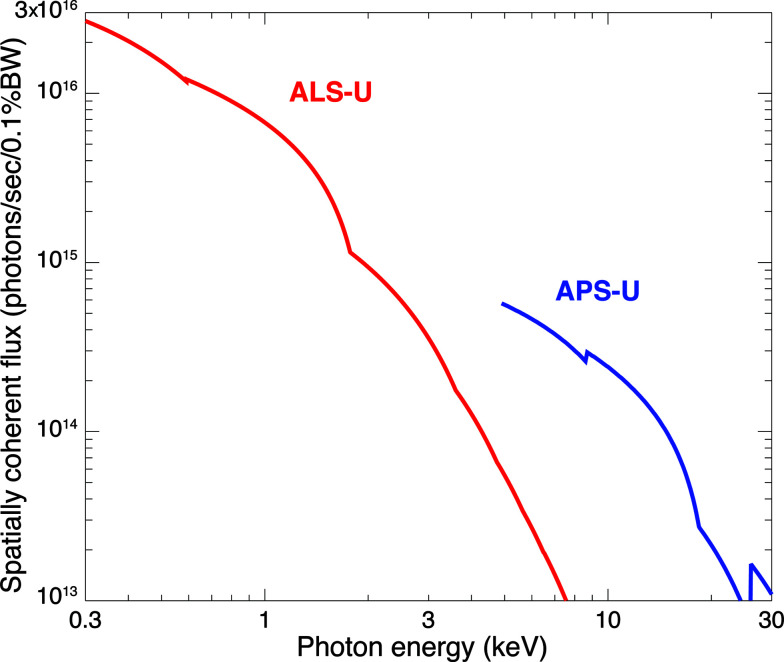
Spatially coherent X-ray flux Φ_c_ that will soon be available from upgrades of the Advanced Light Source at Berkeley (ALS-U) and the Advanced Photon Source at Argonne (APS-U, with high brightness undulators available at *E* ≥ 4.9 keV). To calculate this, we first used the highest value of brightness expected at each particular photon energy, choosing at that energy the best of several planned undulators. The brightness is then multiplied by λ^2^ to give spatially coherent flux within a bandwidth of 0.1%, following common convention, even though the full spectral width of the tunable emission from these undulators is actually closer to 1% (so that approximately ten times higher spatially coherent flux is available). The APS-U involves a shutdown of the storage ring planned for 2022 so as to install a multi-bend achromat lattice for more than a hundredfold gain in hard X-ray brightness (Banks, 2019 ▸). The ALS-U upgrade is likely to follow soon afterwards. APS-U data were provided by Roger Dejus and Michael Borland, while ALS-U data were provided by Christoph Steier.

As an example of the spatially coherent flux Φ_c_ that will soon be available from synchrotron light sources, we show in Fig. 6 ▸ the values anticipated to be available from the ALS-U and the APS-U, multi-bend achromat storage ring lattice upgrades of the Advanced Light Source at Lawrence Berkeley National Laboratory and the Advanced Photon Source at Argonne National Laboratory. This was calculated from the highest value of brightness anticipated from each of several candidate undulators at each facility, rather than from a single example undulator. The spatially coherent flux is conventionally calculated for 0.1% spectral bandwidth, whereas the actual bandwidth of these undulator-based sources is more typically about 1% (thus giving roughly ten times higher spatially coherent flux). While most X-ray beam delivery systems (beamlines) at synchrotron light sources use crystal monochromators with approximately 0.01% bandwidth, which would further reduce the flux compared with 0.1% bandwidth, ptychography can use broader bandwidth for more flux (Enders *et al.*, 2014 ▸), with improved methods being developed for increased-bandwidth ptychographic image reconstruction (Yao *et al.*, 2019 ▸). Thus one can carry out high-throughput ptychography using nanofocused beams (Jacobsen *et al.*, 2017 ▸) by using optics such as ∼1% spectral bandpass multilayer-coated Kirkpatrick–Baez mirrors as have been demonstrated at the ESRF in France (da Silva *et al.*, 2017 ▸).

### 4.1. Idealized per-pixel imaging times   

**Figure 7:**
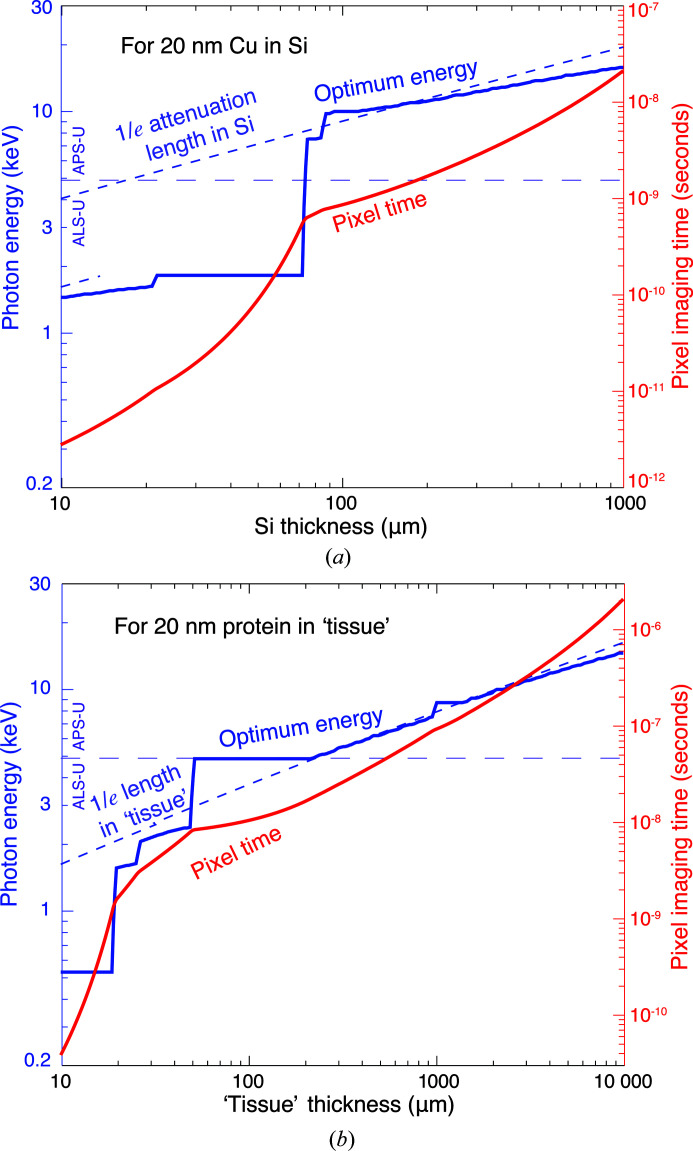
Optimum photon energy *E*_*t*_ (left scale; blue) and per-pixel imaging time *T*_p_ (right scale; red) for imaging 20 nm Cu features in Si (*a*) and 20 nm protein features in a 70% ice/30% protein mixture as tissue (*b*). The pixel imaging time *T*_p_ was calculated according to equation (10), using values of the estimated number of photons 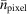 for a variety of photon energies shown in Fig. 2 ▸ and the future spatially coherent flux (at 0.1% bandwidth) values shown in Fig. 6 ▸ for the ALS-U (below 4.9 keV) and the APS-U (at 4.9 keV and above). For each background material thickness value *t*, the smallest value of *T*_p_ is used along with its associated photon energy *E*_*t*_. This per-pixel imaging time *T*_p_ is assumed to be equal to the per-voxel imaging time *T*_v_ due to dose fractionation as discussed in Section 3.1. Also shown is the energy *E*_est_ found by setting μ^−1^(*E*_est_) of equation (4) equal to the total thickness *t* of the background material. In practice, one might be able to accept 1% spectral bandwidth and thus reduce the pixel time by a factor of ten, while reflection efficiencies of beamline and nanofocusing optics might increase the pixel time by about a factor of ten. Therefore the pixel time shown here represents a reasonable estimate.

We now consider the combination of the X-ray brightness *B* soon available, its relationship with the spatially coherent flux Φ_c_ in equation (9) (and as shown in Fig. 6 ▸), and the estimated minimum number of photons per pixel 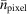 for a variety of photon energies *E* as given by equation (2) and as shown in Fig. 2 ▸. These parameters yield an estimate for a minimum per-pixel imaging time *T*_p_ of 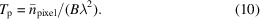
All of the individual terms in equation (10) depend on photon energy *E*. Therefore, rather than use the minimum value of 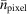 shown (along with the photon energy *E*_*n*_ where 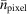 is minimized) in Fig. 4 ▸, we use the set of values of *T*_p_ at all photon energies *E* (as shown in Fig. 2 ▸), and the set of spatially coherent flux values shown in Fig. 6 ▸, to generate a list of candidate pixel times *T*_p_ at all photon energies *E* for each value of background material thickness *t*. On the basis of the considerations of Section 2.3, we can restrict the dose imparted to a subset of results to 10^9^ Gy. For the remaining subset, we then show in Fig. 7 ▸ the minimum pixel time *T*_p_ and the photon energy *E*_*t*_ at which this minimum is obtained. Because of the discontinuity in available coherent flux between the ALS-U below 4.9 keV and the APS-U at 4.9 keV and above, Fig. 7 ▸ shows an inflection point at 4.9 keV in per-pixel imaging time and a discontinuity in the optimum photon energy *E*_*t*_ to use as a function of specimen thickness *t*.

The values of per-pixel time *T*_p_ shown in Fig. 7 ▸ for δ_r_ = 20 nm are for the 0.1% spectral bandwidth conventionally used in light source brightness calculations. However, as noted above, one might be able to accept 1% spectral bandwidth and thus reduce the pixel time by a factor of ten from what is shown in Fig. 7 ▸. At the same time, X-ray beamlines at synchrotron light sources usually use one Kirkpatrick–Baez pair of beamline optics to deliver the illumination to a secondary source position, after which nanofocusing optics can be used to generate the probe wavefield used in ptychography. The combined efficiency of these four optics might be as low as 10% in many implementations. Thus we will assume that the calculation shown in Fig. 7 ▸ is indeed a reasonable representative of achievable per-pixel *T*_p_ and per-voxel *T*_v_ imaging times.

It is obvious that the *T*_p_ values shown in Fig. 7 ▸ are impractically small for conventional approaches using a move–settle–expose or ‘step scan’ method. They should instead be thought of as cumulative times for delivering the required number of photons to an area of 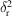 within each object slice shown in Fig. 5 ▸. Strategies for illuminating the specimen will be discussed in Section 5.3 and in Section 2 of the supporting information.

### 4.2. Total imaging times   

**Figure 8:**
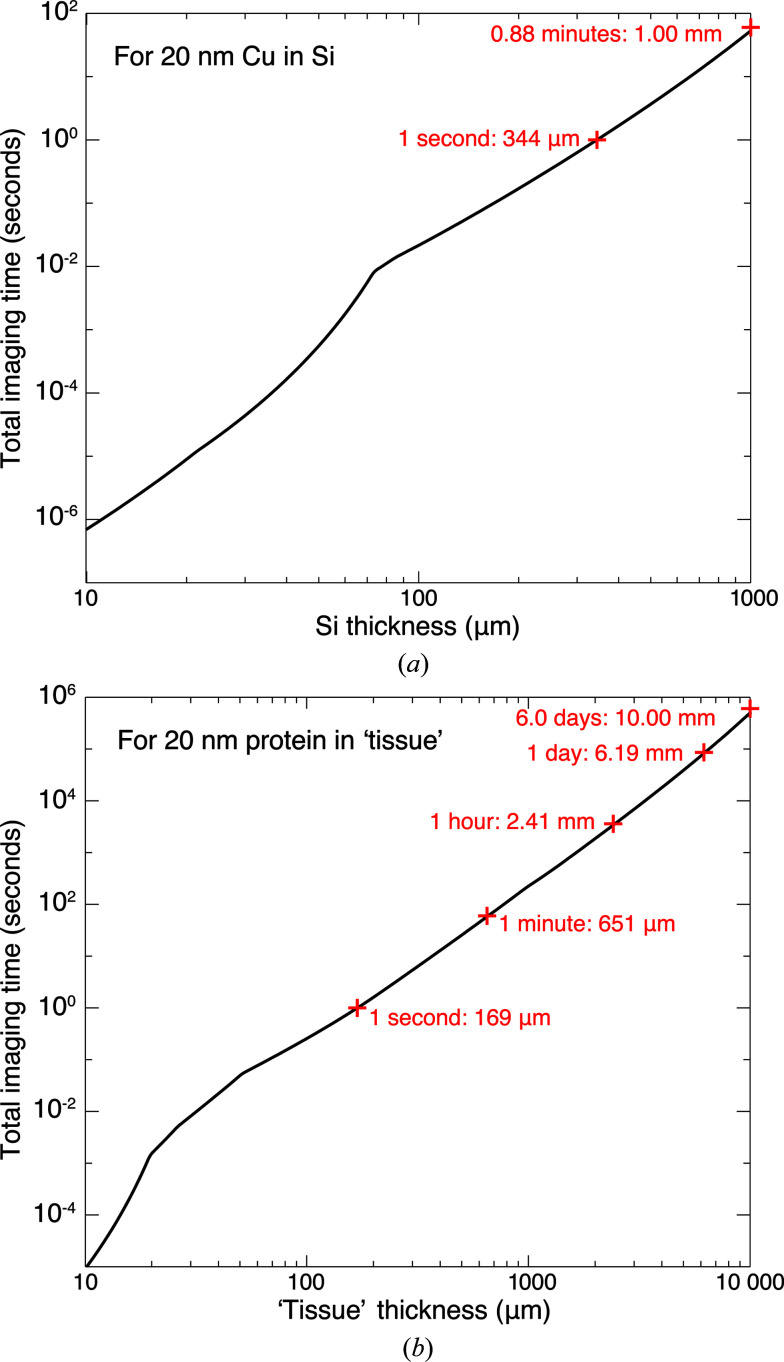
Total time for δ_r_ = 20 nm-resolution imaging of copper features in silicon (*a*) and protein features in 30% protein/70% water tissue (*b*) as a function of specimen thickness *t*. This estimate uses the per-pixel imaging time of Fig. 7 ▸ as input to the calculation of equation (12). The time estimate includes no allowance for ‘dead time’ in the imaging process or any inefficiency losses in the imaging process.

In equation (8) we found that the total number of photons 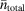 required to image the 3D object is 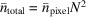. This is equivalent to saying that the total time for imaging *T*_tot_ is equal to the per-pixel imaging time *T*_p_ multiplied by *N* ^2^, or 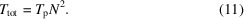
The per-pixel imaging time *T*_p_ was given in equation (10) and is shown in Fig. 7 ▸ along with the photon energy *E*_*t*_ which minimized it. The combination of equations (11) and (10) allows one to calculate the idealized total time *T*_tot_ to image cylindrical specimens with diameter *t* and height *t* as 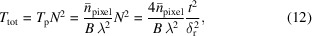
where the last expression uses equation (6). This time is shown in Fig. 8 ▸ for δ_r_ = 20 nm-resolution imaging of copper features in silicon and protein features within tissue consisting of 30% protein/70% ice, at a signal-to-noise ratio of SNR = 5.

## 5. Imaging large specimens: practicabilities   

We now consider some of the other challenges in imaging macroscopic specimens at nanoscale spatial resolution.

### 5.1. Imaging beyond the depth-of-focus limit   

Lens-based imaging involves a depth of focus DOF of (Born & Wolf, 1999 ▸; Jacobsen, 2020 ▸)
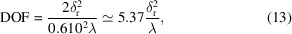
and a similar wave propagation effect applies to coherent diffraction imaging methods such as ptychography. At 15 keV, one has DOF = 6.5 cm with δ_r_ = 1 µm so that one easily obtains pure projection images as required for conventional microtomography, but at δ_r_ = 100 nm one has DOF = 650 µm and at δ_r_ = 10 nm one has DOF = 6.5 µm. Therefore it becomes increasingly necessary to deal with wavefield propagation effects as one improves the transverse spatial resolution δ_r_ for nanoscale imaging of macroscopic objects. Fortunately it is easy to model forward wave propagation through thick complex objects using the multislice method (Cowley & Moodie, 1957 ▸). One can build the multislice method into ptychography reconstruction algorithms (Maiden, Humphry & Rodenburg, 2012 ▸) and thus obtain a series *N*_A_ of axial planes each separated by a depth of focus, so that 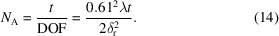
Equation (13) was used for the second form of this expression. Multislice ptychography was first demonstrated using visible light (Maiden, Humphry & Rodenburg, 2012 ▸) and has subsequently been applied to X-ray ptychography (Suzuki *et al.*, 2014 ▸; Tsai *et al.*, 2016 ▸; Öztürk *et al.*, 2018 ▸). One approach is to combine this set of planes and synthesize a pure projection image for use in a standard tomography reconstruction algorithm (Li & Maiden, 2018 ▸). However, one can recover feature detail in the ‘in between’ regions separated by a fraction of a DOF, since the transfer function for most imaging methods has some axial extent (Ren *et al.*, 2020 ▸). Therefore a more accurate approach is to treat beyond-DOF image reconstruction as a numerical optimization problem. In this approach, one begins with a guess of the 3D object (such as that obtained from a conventional 3D reconstruction). For each viewing angle, multislice propagation is used to calculate the wave exiting the present guess of the 3D object, after which one calculates the corresponding expected signal from that angle. This signal might be what is recorded by a conventional imaging system (Van den Broek & Koch, 2012 ▸; Ren *et al.*, 2020 ▸), or a set of far-field coherent diffraction patterns from different illumination angles in diffraction microscopy or Fourier ptychography (Kamilov *et al.*, 2015 ▸; Kamilov *et al.*, 2016 ▸), or a set of far-field coherent diffraction patterns from small, shifted illumination spots in ptychography (Maiden, Humphry, Sarahan *et al.*, 2012 ▸; Tsai *et al.*, 2016 ▸; Gilles *et al.*, 2018 ▸; Du, Nashed *et al.*, 2020 ▸). One then constructs a cost function which is the difference between the expected and observed signals, and minimizes that cost function (while also possibly including additional constraints as regularizers) so as to converge upon an accurate guess of the actual 3D object. Thus, imaging beyond the depth-of-focus limit is possible.

### 5.2. Reducing the number of illumination angles   

**Figure 9:**
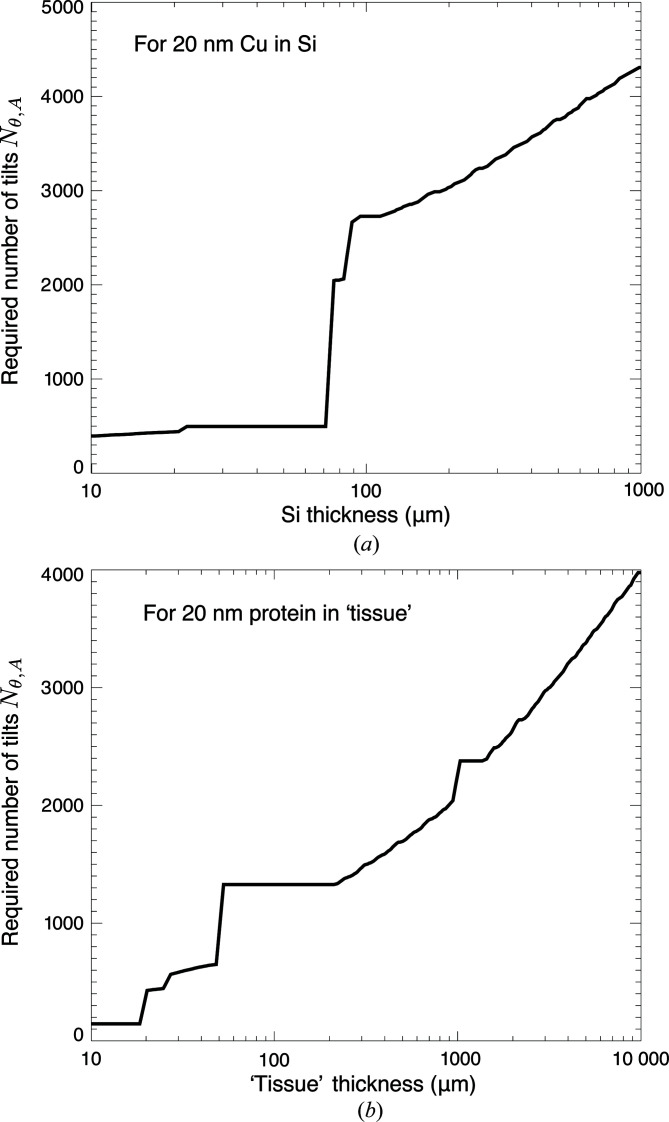
Number of tilts *N*_θ,A_ of equation (15) required for complete coverage of information in the Fourier transform representation of the specimen. Values of *N*_θ,A_ are shown for δ_r_ = 20 nm-resolution imaging of copper features in silicon (*a*) and protein features against ice with an overall thickness of tissue consisting of 30% protein/70% ice (*b*). The number of tilts *N*_θ,A_ is smaller than what would be required to meet the Crowther criterion *N*_θ_ [equation (7)] when reconstructing *N*_A_ axial planes in beyond-depth-of-field imaging (Jacobsen, 2018 ▸). For each sample thickness *t*, the photon energy *E*_*t*_ that minimizes the per-pixel imaging time *T*_p_ was used in calculating *N*_θ,A_; this photon energy *E*_*t*_ is shown in Fig. 7 ▸.

In Section 3.2, it was noted that complete coverage of information in the 3D Fourier transform of an object requires that one acquires projection images over *N*_θ_ = (π/2)*N* tilt angles [equation (7)], with this requirement known as the Crowther criterion (Crowther *et al.*, 1970 ▸). This applies to pure projection images, which convey no information on the location of features along the projection direction (so that the *N* × 1 pixel image of an object slice yields *N* × 1 pixels in the Fourier transform). If, however, wavefield propagation provides that information so that one reconstructs images at each of *N*_A_ axial planes, one has information over *N* × *N*_A_ pixels in the Fourier transform, so that a complete filling of information at the outer circumference involves not *N*_θ_ but *N*_θ,A_ = *N*_θ_/*N*_A_ rotation angles (Jacobsen, 2018 ▸), a relationship that is consistent with subsequent experimental results (Tsai *et al.*, 2019 ▸; Huang *et al.*, 2019 ▸). From equations (7), (6) and (14), one can write the required number of angles *N*_θ,A_ for complete information in the Fourier plane as 
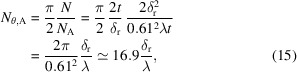
which surprisingly does not depend on the overall sample size *t*. However, the optimum photon energy *E*_*t*_ (and thus the wavelength λ) for minimizing the per-pixel imaging time *T*_p_ does change with sample thickness *t*, as shown in Fig. 7 ▸. Therefore we show in Fig. 9 ▸ the required number of tilts versus *t* as obtained using the optimum wavelength λ = *hc*/*E*_*t*_ for each thickness.

### 5.3. Ptychographic imaging considerations   

As noted in Section 2.4, ptychography is a dose-efficient imaging method, since no potentially lossy optics are placed between the specimen and the detector, and one can use efficient direct X-ray detection in pixel array detectors with large pixel size. However, since it involves the collection of a set of diffraction patterns from a finite-sized coherent beam (the probe) placed at a set of probe positions, rather than the collection of a full image field in one exposure, one must consider ways to maximize its throughput. This is discussed in Section 2 of the supplementary material, which discusses several approaches to dramatically increasing the throughput of X-ray ptychography towards what is needed to realize the per-pixel exposure times shown in Fig. 7 ▸.

## 6. Highlighting features of interest   

Previous imaging time estimations are for imaging all voxels with identical dose, assuming intrinsic contrast of nanoscale features in two example specimens. We now discuss two ways to potentially increase imaging throughput: by increasing the contrast of specific features through staining (in the case of biological specimen preparation), and by ‘smart sampling’ using artificial intelligence approaches in data acquisition.

### 6.1. Connectomics: to stain or not to stain?   

Although phase contrast imaging of unstained samples yields good contrast, its biological interpretability compared with stained samples is an open topic. In biological imaging, the structural complexity of specimens and the minute gradual spatial variations of refractive index frequently motivate the use of stains to selectively enhance contrast. The staining process introduces extrinsic chemical compounds into the specimen to highlight specific features against the background [such as membranes relative to the cytosol in the context of connectomics, so as to delineate cellular boundaries; Mikula & Denk, 2015 ▸; Hua *et al.*, 2015 ▸). The modification of the mol­ecular content in the tissue microenvironment is achieved either via physical aggregation or via chemical binding of histological dyes or immunohistochemical agents to macromolecules (Prentø, 2009 ▸). The challenge is to provide sufficient contrast enhancement for desired features without unduly increasing overall absorption in thick specimens. If overall absorption were to be increased significantly through staining, higher photon energies would be needed to maintain transmission through the specimen. Therefore, one needs to evaluate the balance between the contrast increase that a stain provides, and the contrast decrease and concomitant increase in required fluence at higher photon energies (as shown in Fig. 2 ▸ in the case of an unstained model specimen). Available coherent flux also decreases at higher energies, as shown in Fig. 6 ▸.

As noted in the supplementary material, X-ray microscopy has been used to study both stained and unstained brain tissue. If information on preparation protocols and resulting image contrast is deposited in publicly available neuroscience databases (Vogelstein *et al.*, 2016 ▸, 2018 ▸), one can better compare approaches across different imaging modalities to help determine the optimal staining method for adopting X-ray microscopy in connectomics.

### 6.2. Needles in a haystack: machine learning for adaptive scanning   

The full-specimen imaging times discussed in Section 4.2 assume equal fluence to all voxels in a 3D specimen. However, this is not always required. Consider the example of Section 1.1, where the goal is to image neuronal cell bodies and processes and, in particular, synaptic connections between them. This is a hierarchical imaging problem (Wacker *et al.*, 2016 ▸; Burnett & Withers, 2019 ▸), with micrometre-scale spatial resolution required to see cell bodies, 100 nm-scale spatial resolution required to see dendritic spines, but perhaps 20 nm spatial resolution required to see if synapses are present at points where two neuronal processes might be proximal. Given that synapses represent a volume fraction of only about 9 × 10^−5^ in mouse brains, and that they are randomly distributed (Anton-Sanchez *et al.*, 2014 ▸), can one use lower voxel fluence on the 99.991% of the mouse brain volume and higher fluence for accurate identification of synaptic connections? Techniques such as Bayesian compressive sensing (Donoho, 2006 ▸; Candès *et al.*, 2006 ▸; Ji *et al.*, 2008 ▸) have been successfully applied to image acquisition (Trampert *et al.*, 2018 ▸; Stevens, Luzi *et al.*, 2018 ▸) and demonstrated real-time feedback during scanning. In subsampled ptychography, one first learns a ‘dictionary’ of textures present in the specimen (Kreutz-Delgado *et al.*, 2003 ▸; Aharon *et al.*, 2006 ▸) and then uses this dictionary to ‘inpaint’ the most likely combination of textures into image regions that have sparsely sampled actual data. This capability is particularly beneficial to applications such as integrated circuits, which have numerous copies of near-identical structures. However, this approach will not work when an axon and dendrite are in close proximity without having an actual synaptic connection (Kasthuri *et al.*, 2015 ▸); that is, one may have regions which look very similar in undersampled data so that the act of inpainting could potentially lead to an unacceptably high number of false (connection) positives in the reconstructed connectome. Therefore, a ‘smart’ scanning is desired, which can adaptively learn a model to optimize the overall dose. One candidate to achieve a high-speed and dose-efficient scan is the ‘active learning’ approach (Cohn *et al.*, 1996 ▸), which enables an adaptive X-ray experimental design that optimally distributes resources (time, tolerable dose *etc*.) and acquires the ‘useful’ data at minimum cost. Active learning frameworks have shown success in many fields (Tong, 2001 ▸), including microbiology (Hajmeer & Basheer, 2003 ▸), neurophysiology (Lewi *et al.*, 2009 ▸) and manufacturing (Jones *et al.*, 2010 ▸). One possible way of introducing active learning to X-ray ptychography experiments would be to train a neural network that predicts the possibility for a scanned region to contain important features and adjusts the dose to be invested into that region accordingly. We provide extended discussion on this point in Section 3 of the supplementary material. With these strategies, the total imaging times shown in Fig. 8 ▸ can be potentially further reduced.

## 7. Related literature   

The following additional literature is referenced by the supporting information: Allahgholi *et al.* (2019 ▸); Bourassa & Miller (2012 ▸); Bunk *et al.* (2008 ▸); Chang & Sakdinawat (2014 ▸); Clarke & Royle (2019 ▸); Dierolf *et al.* (2010 ▸); Dwivedi *et al.* (2018 ▸); Edo *et al.* (2013 ▸); Fera *et al.* (2020 ▸); Genoud *et al.* (2018 ▸); Guizar-Sicairos *et al.* (2014 ▸); Gürsoy (2017 ▸); Heuser & Reese (1981 ▸); Heuser *et al.* (1979 ▸); Huang *et al.* (2014 ▸, 2015 ▸, 2017 ▸); Jacobsen *et al.* (1991 ▸); Jefimovs *et al.* (2007 ▸); Jin *et al.* (2017 ▸); Kaestner *et al.* (2011 ▸); Kamaya *et al.* (2011 ▸); Kavalali & Jorgensen (2014 ▸); Khimchenko *et al.* (2016 ▸); Kim *et al.* (2005 ▸); Kirz (1974 ▸); Lam *et al.* (2015 ▸); Li *et al.* (2020 ▸); Martin & Koch (2006 ▸); McAllum & Hare (2019 ▸); Mohacsi *et al.* (2015 ▸, 2017 ▸); Moor (1987 ▸); Munnig Schmidt (2012 ▸); O’Toole *et al.* (1993 ▸); Perrin *et al.* (2015 ▸); Ren *et al.* (2016 ▸); Sang *et al.* (2016 ▸); Sayre *et al.* (1977 ▸); Schneider (1997 ▸); da Silva & Menzel (2015 ▸); Stevens, Yang *et al.* (2018 ▸); Sullivan *et al.* (2014 ▸); Thibault & Menzel (2013 ▸); Uhlén *et al.* (2014 ▸); Velazco *et al.* (2020 ▸); Victor *et al.* (2020 ▸); Watanabe *et al.* (2013 ▸, 2014 ▸); Wilke (1983 ▸); Zhang *et al.* (2019 ▸); Ziegler *et al.* (2017 ▸).

## 8. Conclusion   

The emergence of diffraction-limited storage rings to continue the historical trend of rapid increases in available coherent X-ray flux allows us to think of a new possibility: extending nanoscale X-ray imaging to macroscopic specimens. The ability of X rays to penetrate thick samples has been recognized since Röntgen’s initial discovery, but nanoscale X-ray imaging has been applied only to microscopic objects. In addition, conceptual and algorithmic advances have been made to overcome the depth-of-focus limit in coherent X-ray imaging and to reduce the number of rotation angles required for full data sampling.

Using a model that gives excellent agreement with thin-specimen observations of the photon fluence required for imaging features of a given contrast and resolution, we have incorporated the corrections required for thick-specimen imaging (Du & Jacobsen, 2018 ▸, 2020 ▸). We have then considered the coherent flux that should be available at various X-ray energies from two example forthcoming diffraction-limited storage ring facilities (the APS at Argonne and the ALS at Berkeley). This has allowed us to calculate the minimum per-pixel imaging time as well as the photon energy that minimizes the imaging time, and extrapolate that to conceivable whole-specimen 3D imaging times.

This analysis has made clear several challenges that should be resolved to fully exploit the worldwide investment in diffraction-limited storage ring light sources. If we use ptychography as a particularly dose-efficient and non-optics-limited imaging approach, we will need dramatic advances in the available frame rate of detectors with a modest number of pixels, such as megahertz frame rates for 256^2^ pixels (on-detector lossy data compression might help in reaching this performance level; Huang *et al.*, 2021 ▸). We will need improved high-speed scanning systems and ‘smart’ scanning systems to potentially adjust the cumulative number of photons used per voxel to collect a larger signal where needed for critical feature identification and a smaller signal from other regions. But even without ‘smart’ scanning approaches, we show that one might ultimately consider imaging millimetre-sized copper-in-silicon specimens in about a minute and centimetre-sized biological specimens in about a week. Nanoimaging of macroscopic specimens is a real possibility for the future.

## Supplementary Material

Supplementary Information. DOI: 10.1107/S1600576721000194/jo5064sup1.pdf
