# Supplementary material for: Upscaling X-ray nanoimaging to macroscopic specimens
Source: J Appl Crystallogr. 2021 Feb 19;54(Pt 2):386–401. doi: 10.1107/S1600576721000194 (PMC8056767; doi:10.1107/S1600576721000194)
Supplement: Supplementary file 1 [file j-54-00386-sup1.pdf]

Supplementary information: upscaling x-ray nanoimaging to macroscopic specimens

Ming Du,[a] Zichao (Wendy) Di,[a,b] Doğa Gürsoy,[a,c] R. Patrick Xian,[d] Yevgenia Kozorovitskiy[d,e] and Chris Jacobsen [a,f,e]*

[a]*Advanced Photon Source, Argonne National Laboratory, Argonne, IL 60439 USA,* [b]*Mathematics and Computer Science Division, Argonne National Laboratory, Argonne, IL 60439 USA,* [c]*Department of Electrical Engineering and Computer Science, Northwestern University, Evanston, IL 60208 USA,* [d]*Department of Neurobiology, Northwestern University, Evanston, IL 60208 USA,* [e]*Chemistry of Life Processes Institute, Northwestern University, Evanston, IL 60208 USA, and* [f]*Department of Physics & Astronomy, Northwestern University, Evanston, IL 60208 USA. E-mail: cjacobsen@anl.gov*

1. Comparison of ptychography with other x-ray nanoimaging approaches

In the main text, we have chosen to consider x-ray ptychography as a dose-efficient imaging method with no optics-imposed limit to spatial resolution. Optics-based full-field x-ray imaging is frequently done using Fresnel zone plates which have a focusing efficiency in theory of up to about 20% (Kirz, 1974; Schneider, 1997), but only about 1–5% in practice for sub-30 nm resolution multi-keV x-ray zone plates (Jefimovs *et al.*, 2007; Uhlén *et al.*, 2014; Chang & Sakdinawat, 2014; Mohacsi *et al.*, 2015; Mohacsi *et al.*, 2017; Li *et al.*, 2020). Another very popular and successful approach for imaging a large field of view is x-ray nanoholography using a nanofocus point source and

geometric magnification onto a scintillator-based detector (Mokso *et al.*, 2007), but the scintillator detectors used often have quantum efficiencies of 20% or lower (Martin & Koch, 2006). If one can instead place inefficient components *upstream* of the specimen being imaged, the inefficiencies increase the overall imaging time. However, they do not lead to the specimen receiving a higher radiation dose. This is the case of scanning microscopes, which require spatially coherent illumination in order to obtain diffraction-limited resolution from the optic (Jacobsen *et al.*, 1991; Jacobsen, 2020).

Scanning and full-field imaging make different demands on instrument stability. Because full-field imaging in absorption or Zernike phase contrast can be done with incoherent sources and with all pixels collected in parallel, it can be done with much shorter exposure times so that there is less sensitivity to position drift between specimen and optic. In x-ray nanoholography, drift can lead to blurring of the image but one does not have to quickly move beam positions as in scanned beam methods. With scanned beam methods, drift during data collection can lead to distortions in the image or resolution degradation in the case of ptychography; however, one can correct for these scanned beam position errors in ptychography (Guizar-Sicairos & Fienup, 2008; Zhang *et al.*, 2013; Dwivedi *et al.*, 2018).

2. Scaling of previous experiments

Scaling from previous experiments can add confidence to the estimates made in the main text.

Two examples of high-throughput 2D ptychographic imaging of the integrated circuit layer within a silicon wafer provide insights on what can be achieved. Using 6.2 keV X rays at the Swiss Light Source to image in a 10 μm thick silicon wafer, an effective imaging rate of $T_p = 40$ μs for a resolution of $\Delta_p = 41$ nm was achieved (Guizar-Sicairos *et al.*, 2014). At the Advanced Photon Source at Argonne, 8.8 keV

X rays were used to image in a 130 µm thick silicon wafer (Deng *et al.*, 2019); a double-multilayer monochromator was used to obtain a spatially coherent flux of about 1×10^{10} photons/s in 1% spectral bandpass, with an imaging rate of $T_p = 11$ µs for a resolution of $\Delta_p = 10$ nm. This latter example involved scanning the focus of a Fresnel zone plate with 50 nm outermost zone width (and thus 49 nm theoretical probe FWHM size, given that the central 33% of the optic was obstructed with a central stop; see Fig. 4.32 of (Jacobsen, 2020)) at a linear speed of 150 µm/s while collecting 3000 diffraction patterns per second. Both of these examples scale reasonably well to the pixel time estimates shown in Fig. 7 of the main text when one accounts for sample thickness, spatial resolution, photon energy used, and coherent flux values obtainable in the future as shown in Fig. 6. In the latter case, the main limitation was the maximum "frame rate" of the detector used. While there are direct-detection x-ray pixel array detectors that use per-pixel analog charge storage to record up to 352 images at a burst frame rate of 4.5 MHz, the sustained frame rate of these detectors is limited to 16 kHz, or a sustained per-frame time of 63 µs (Allahgholi *et al.*, 2019).

The Swiss Light Source example (Guizar-Sicairos *et al.*, 2014) can be scaled to larger sample imaging as follows:

- This example was done at the present-day Swiss Light Source with a brightness about 600 times lower than that expected from the APS-U (the APS-U brightness was used in calculating the spatially coherent flux shown in Fig. 6 of the main text; it should be noted that the Swiss Light Source is also planning an upgrade to a diffraction-limited storage ring). Thus one can divide the exposure time by 600 to account for this.
- The experiment at the C-SAXS beamline used a double-silicon-crystal monochromator with about 0.01% spectral bandpass. In fact, one can carry out ptychography using a much broader bandwidth (Enders *et al.*, 2014; Yao *et al.*, 2019),

such as the full 1% bandwidth available from typical undulator sources. Thus one can divide the exposure time by 100 for (1% bandpass/0.01% bandpass).

- The experiment at the C-SAXS beamline used a Fresnel zone plate focusing optic with 100 nm thick gold zones with a theoretical diffraction efficiency (Kirz, 1974) of about 0.6%, whereas broad bandpass multilayer-coated Kirkpatrick-Baez mirror optics have demonstrated 45% focusing efficiency (da Silva *et al.*, 2017). These optics were used in connection with a multilayer-coated monochromator with 4% bandpass so as to limit exposure to other photon energies, so it is reasonable to assume a combined efficiency for monochromator and optics of about 20%. Thus one can divide the exposure time by (20%/0.6%), or a factor of 33.
- The C-SAXS experiment was done at 41 nm spatial resolution, whereas the pixel time calculation of Fig. 7 of the main text was for 20 nm resolution. We use the scaling of $\bar{n}_{\text{pixel}} \propto t_f^{-2}$ of Eq. 2 of the main text to translate the results to higher spatial resolution. Thus one must multiply the exposure time by $(41/20)^2 = 4.2$.

This leads to an estimated per-pixel imaging time of 8.4×10^{-11} seconds using 6.2 keV photon energy. In fact, the pixel time calculation shown in Fig. 7 of the main text suggests that the optimum photon energy to use for 10 μm thick silicon background is 1.45 keV rather than 6.2 keV, so let us also consider the scaling of this result to 1.45 keV photon energy:

- From the data used in Fig. 6 of the main text, an ALS-U undulator is expected to provide a coherent flux of 30.8×10^{14} photons/s at 1.45 keV, while an APS-U undulator is expected to provide 4.45×10^{14} photons/s at 6.2 keV. Thus one can divide the exposure time by (30.8/4.45)=6.9.
- From Eq. 2 of the main text, we see that the contrast scaling s_c of the required number of photons $\bar{n}_{\text{pixel}}$ per pixel due to changes in image contrast when moving

from λ_1 to λ_2 goes as

$$\begin{aligned} s_c &= \left(\frac{\lambda_2}{\lambda_1}\right)^2 \frac{|\delta_{f,1}-\delta_{b,1}|^2}{|\delta_{f,2}-\delta_{b,2}|^2} = \left(\frac{E_1}{E_2}\right)^2 \frac{|\delta_{f,1}-\delta_{b,1}|^2}{|\delta_{f,2}-\delta_{b,2}|^2} \\ &= \left(\frac{1.45\ \text{keV}}{6.2\ \text{keV}}\right)^2 \frac{|7.494\times 10^{-4}-1.995\times 10^{-4}|^2}{|4.255\times 10^{-5}-1.282\times 10^{-5}|^2} = 18.7 \end{aligned} \quad (1)$$

where we have used tabulated values (Henke *et al.*, 1993) for the refractive index of copper and silicon at 1.45 keV versus 6.2 keV. Thus one can divide the exposure time by 18.7.

- Also from Eq. 2 of the main text, we must correct for the increased absorption in the 10 μm silicon background thickness when moving from λ_1 to λ_2. This absorption scaling s_a goes as

$$s_a = \frac{\exp[\mu_2 t_{b\prime}]}{\exp[\mu_1 t_{b\prime}]} = \frac{\exp\left[(7.41\ \mu\text{m})^{-1}\cdot(10\ \mu\text{m})\right]}{\exp\left[(33.5\ \mu\text{m})^{-1}\cdot(10\ \mu\text{m})\right]} = 2.9 \quad (2)$$

using tabulated values (Henke *et al.*, 1993) for the linear absorption coefficient μ of silicon. Thus one must multiply the exposure time by 2.9 to account for the absorption scaling s_a.

Taken together, the scaling of this example to 14.5 keV photon energy leads to an estimate of a per-pixel imaging time of 6.5×10^{-13} seconds, whereas the calculation results shown in Fig. 7 of the main text give a result of 28.4×10^{-13} seconds for the per-pixel imaging time. Given the nature of the estimates that the above scaling factors involve, this discrepancy of a factor of 4.4 is not outlandish.

These examples indicate that significant advances in ptychography methods and instrumentation will be required to scale x-ray nanoimaging up to macroscopic objects:

- One of the adjustable parameters in a ptychographic imaging experiment is the ptychographic resolution gain

$$G_p = \frac{d}{\delta_\text{r}} \quad (3)$$

which is the ratio of the diameter d of the scanned coherent illumination spot relative to the target spatial resolution δ_r (for simplicity, we assume a circular

illumination spot, though in practice one might have an Airy illumination pattern from a lens). In order to maximize the ptychographic imaging throughput, small values of G_p are preferred (Jacobsen *et al.*, 2017). With a smaller ptychographic gain G_p, one can relaxed the spectral bandwidth $\Delta\lambda/\lambda \leq 2/G_p$ and thus make better use of the full $\sim 1\%$ bandwidth of the output of undulators at diffraction-limited storage rings (Fig. 6 of the main text). The minimum number of pixels $N_{\mathrm{det,min}}$ required along one edge of the detector is

$$N_{\mathrm{det,min}} = 2G_p, \tag{4}$$

while the exposure time per illumination spot T_p can be written as

$$T_p = \frac{N_0 G_p^2 (1 - f_d)^2}{I_0} \tag{5}$$

in order to achieve a per-pixel fluence of $N_0/\delta_{\mathrm{r}}^2$. The expression of Eq. 5 involves the ptychographic overlap fraction f_d, which (in the case of uniform illumination over a circular diameter d) is the distance over which the illumination spot is moved relative to d, with values of $f_d \lesssim 0.5$ preferred (Bunk *et al.*, 2008; Deng *et al.*, 2015; Huang *et al.*, 2017). (One can relax this 2D position overlap requirement when using angular overlap in tomography experiments (Gürsoy, 2017), but it remains an open question whether this is still the case when one also relaxes the Crowther criterion for the number of illumination angles as discussed in Sec. 5.2 of the main text). Smaller values of ptychographic gain G_p also decrease the dynamic range required by the detector (Jacobsen *et al.*, 2017). Thus the ideal detector for high throughput ptychography is one with a relatively modest number of pixels $N_{\mathrm{det,min}}$ (for example, 128×128 or 256×256 pixels) but the highest possible frame rate, ideally approaching the inverse of the per-pixel illumination time shown in Fig. 7 of the main text.

- A standard ptychographic dataset with a 2D diffraction pattern per 2D probe

position provides information compartmentalized in 4 dimensions, so that it is highly redundant for reconstructing a 2D complex exit wave from the specimen (Edo *et al.*, 2013; da Silva & Menzel, 2015). One can therefore use sparse sampling and regularizers in optimization-based image recovery approaches, as discussed in Sec. 5.1 of the main text, to obtain successful ptychographic image reconstructions with compressed data (Stevens *et al.*, 2018*b*).

- The method originally employed for continuous-probe-motion ptychography was a simple raster scan with a sawtooth position-versus-time scan in one direction, and a discrete position step in the orthogonal transverse direction (Pelz *et al.*, 2014; Deng *et al.*, 2015; Huang *et al.*, 2015). However, other scanning approaches have been developed, including spiral scans for reducing artifacts with reduced probe overlap (Dierolf *et al.*, 2010; Huang *et al.*, 2014). Archimedean spiral scans have been used for high-speed scanning in atomic force and scanning tunneling microscopy (Sang *et al.*, 2016; Ziegler *et al.*, 2017), and Hilbert "snake" scans have been used in scanning transmission electron microscopy (Velazco *et al.*, 2020). Lissajous trajectories offer another approach to minimize sharp accelerations in both orthogonal scanning directions (Sullivan *et al.*, 2014). One can also use specimen rotation as the fast axis for ptychography (Gürsoy, 2017). Therefore, one can use some flexibility in choosing a scanning approach that best exploits the capabilities, and avoids imposed limitations, of the scanning stage and control system at hand.
- Because x-ray mirrors operate at grazing incidence, it is difficult to use methods such as the resonant galvanometric mirror pairs of confocal light microscopy (Wilke, 1983) to scan the illumination beam. Instead, one usually physically scans either the specimen or the optic. If the optic is scanned, one risks translating the coherent illumination variations on the optic into variations on the

complex illuminating probe. While one can account for probe variations in the reconstruction (Thibault & Menzel, 2013), one can also avoid this by using rapid optic scanning over small areas and slower specimen scanning over larger areas (Deng *et al.*, 2019). While rapid, precise scanning is technically challenging, the challenges can be met if sufficient resources are brought to bear. This is most starkly evident in the example of wafer scan systems for lithographic fabrication of integrated circuits, where position accuracies at the nanometer level have been obtained with scan velocities in the meter per second range, and accelerations of tens of meters per second squared (Munnig Schmidt, 2012).

3. Smart scanning approaches

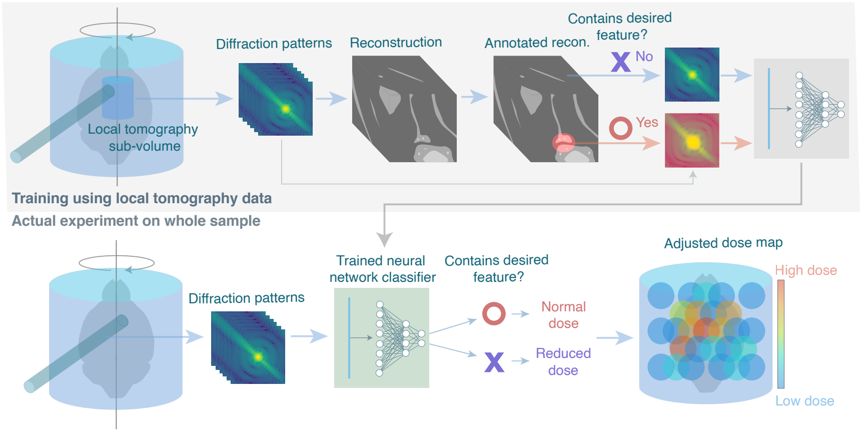

Fig. 1. Schematic of a possible implementation of neural network-based adaptive scanning for x-ray ptychotomography. By doing local ptychotomography on a sub-volume of the sample (top figure), one can annotate features in the reconstructed image and use them to train a neural network to identify diffraction patterns that are obtained when the beam crosses a desired feature (such as a synapse in brain tissue). When scanning the entire sample at low fluence (with beam positions shown as blue dots in the lower figure), the trained neural network allows one to identify regions which are likely to contain the desired features, and thus increase the x-ray fluence used (shown as orange or red illumination spots in the lower figure) either on this scan or on a subsequent scan at a slightly different angle in tomographic data collection.

As noted briefly in the main text, one can improve imaging speed using "smart scanning" to collect more data from crucial regions (such as synapses in brain tissue), and less data from homogeneous regions, or regions which do not contain information that is as crucial for understanding the material under study.

For some types of specimens, unsupervised machine learning can be applied directly on incrementally acquired data. This allows one to extract distinct features in the sample, and thus guide scanning trajectories to regions of scientific interest. Techniques such as Bayesian compressive sensing (Donoho, 2006; Candès *et al.*, 2006; Ji *et al.*, 2008) have been successfully applied to image acquisition (Trampert *et al.*, 2018; Stevens *et al.*, 2018*a*) and demonstrated real-time feedback during scanning. In subsampled ptychography, one first learns a "dictionary" of textures present in the specimen (Kreutz-Delgado *et al.*, 2003; Aharon *et al.*, 2006) and then uses this dictionary to "inpaint" the most likely combination of textures into image regions that have sparsely sampled actual data. This capability is particularly beneficial to applications such as integrated circuits that have numerous copies of near-identical structures. However, such approach will not work when an axon and dendrite are in close proximity without having an actual synaptic connection (Kasthuri *et al.*, 2015); that is, one may have regions which look very similar in undersampled data so that the act of inpainting could potentially lead to an unacceptably high number of false (connection) positives in the reconstructed connectome. Therefore, a "smart" scanning is desired which can adaptively improve its own scanning strategy.

One candidate to achieve the high-speed and dose-efficient scan is through the so-called "active learning" approach (Cohn *et al.*, 1996) which enables an adaptive x-ray experimental design that optimally distributes resources (time, tolerable dose, etc.) and acquires the "useful" data at minimum cost. Active learning frameworks have shown successes in many fields (Tong, 2001) including microbiology (Hajmeer

& Basheer, 2003), neurophysiology (Lewi *et al.*, 2009), and manufacturing (Jones *et al.*, 2010). Their capability to automatically identify and classify sample features will allow real-time informative feedback, and can be the key to studies of small features in a large sample volume. Furthermore, an active learning approach can provide a structured and amendable framework to incorporate *a priori* morphological knowledge into the data acquisition protocol.

Another application is the detection of the cracks in battery materials, since these cracks indicate degradation of the material leading to lower energy density and higher product-life-cycle cost (Kamaya *et al.*, 2011). Here one wishes to catch rare events such as crack formation and propagation that occur over sub-second timescales, with nanometer-scale detail yet with cracks that may extend to lengths of millimeters. Traditional full-specimen raster scanning schemes will miss the details of crack formation during the charging process. Instead, intelligent scanning schemes that dynamically guide data collection along the crack as it forms in *operando* experiments can allow one to trace crack formation and evolution, hopefully leading to an understanding of the underlying mechanisms and contributing to the development of improved battery materials. In this context, initial random coarse scanning of a large region can first provide a refined region of interest, and rapidly train a neural network to recognize crack features in either direct (real-space) or indirect (e.g., ptychographic) imaging. This can then guide fine scanning along the developing crack, with additional data being used to further refine the learned model and possibly incorporate additional evolving features.

Similarly, one could devise a different type of "smart" scanning to optimize the fluence. For example, one can use local tomography to image a small volume at uniform full fluence, and carry out a detailed analysis on that volume to identify the subregions where full fluence is actually required for unambiguous feature detection. In the con-

text of ptychography, one could only acquire the coherent diffraction patterns obtained from illuminating the previously identified critical features, and use these data to train a neural network to recognize the regions where higher fluence is required to recognize more structural detail. When scanning the entire specimen, one could use the 2D scan taken at one rotational angle to inform the scanning hardware at the next rotation angle on how to modulate the scanning speed to adjust the fluence (see Fig. 1). In a rotation-as-fast-axis ptychographic approach (Gürsoy, 2017), one could use fast modulation of the transverse probe position during rotation to achieve a similar result. In the case of connectomics, this could let one go from lower fluence data telling one where synapses *might* be, to acquire sufficient signal for high confidence in knowing that a specific region is (or is not) a synapse.

A challenge that this "smart" scanning scheme may face is that when a thick specimen is imaged, each 2D diffraction pattern encodes the projected information through the sample, and the feature overlay along the beam axis, as mentioned at the beginning of Sec. 3 of the main text, may hinder the neural network from determining exactly whether the diffraction pattern contains the desired feature. A possible workaround is to use a stack of diffraction patterns collected from multiple (preferably largely spaced) viewing angles in both training and prediction, so that the multi-angle input can hopefully disambiguate the projection overlay. In practice, this solution would be more compatible with the rotation-as-fast-axis ptychographic approach if multi-angle diffraction patterns are acquired in an interleaving fashion (Kaestner *et al.*, 2011): for each lateral probe position, one could first collect a few diffraction patterns covering the full range of rotation, and pass the data to the neural network. This could allow the neural network to predict whether the ring-shaped region contains the desired feature, and if not, subsequent diffraction patterns at interleaving angles can be acquired with lower fluence.

4. To stain, or not to stain: examples in the literature

As noted in the main text, tissue staining methods offer improved contrast but reduced penetration. Several *en bloc* staining protocols have been developed for osmium and uranium staining of membranes deep into brain tissue (Mikula & Denk, 2015; Hua *et al.*, 2015; Genoud *et al.*, 2018), as required for serial sectioning and serial blockface imaging in electron microscopy connectomics. These protocols typically require significant time to allow for stain infiltration, and they can show variability in staining depth and staining density (Fera *et al.*, 2020). Both challenges increase with specimen size. An alternative approach is to use immunohistochemical (IHC) methods engineered to target different receptors of the neurons and extraneuronal tissue based on antibody binding selectivity. Their recent use for macroscopic x-ray imaging demonstrates protocol scalability to the entire mouse brain (Depannemaecker *et al.*, 2019; Massimi *et al.*, 2019), though ultrastructural integrity has yet to be evaluated in detail. Molecular tags developed for correlative cellular imaging (Clarke & Royle, 2019; Victor *et al.*, 2020) might also prove useful for staining brain specimens. Alternatively, using genetic labeling approaches, specific heavy or exogenous elements can also be transported to target location via adeno-associated virus following *in vivo* injection (Zhang *et al.*, 2019).

From an imaging perspective, the staining and labeling methods described above modify the spatially-resolved absorptive (β) and phase-shifting (δ) parts of the x-ray refractive index simultaneously (Eq. 1 in the main text), so as to increase contrast. Unfortunately, the chemical modification of stains and fixatives can lead to alteration of the specimen morphology at the subcellular level (O'Toole *et al.*, 1993). In light of this, we may ask the question whether absorption or phase contrast of unstained brain specimens alone is sufficient for delineating structural boundaries for connectomics. There are already examples of x-ray imaging of unstained brain tissue where

certain neuronal features can be identified, including examples with paraffin embedding (Khimchenko *et al.*, 2016; Hieber *et al.*, 2016; Ren *et al.*, 2016; Töpperwien *et al.*, 2017; Khimchenko *et al.*, 2018).

Because the radiation dose necessarily imparted to a specimen increases significantly with improvements in spatial resolution (Sayre *et al.*, 1977; Howells *et al.*, 2009), cryogenic specimen preparation and imaging conditions are preferred for preserving morphology (O'Toole *et al.*, 1993) and chemistry (Perrin *et al.*, 2015; Jin *et al.*, 2017). Present-day cryogenic sample preparation aims mainly at the smaller specimen sizes amenable to electron microscopy. High-pressure freezing has been developed mainly for sub-millimeter-size specimens (Moor, 1987) but approaches based on gas pressurization followed by cooling (Kim *et al.*, 2005) are being explored for cryogenic sample preparation of larger specimens. Cryogenic imaging has used for studies of chemically fixed but unstained brain tissue (Shahmoradian *et al.*, 2017), and also paraffin-embedded, stained brain tissue (Kuan *et al.*, 2020). The latter study compared phase contrast x-ray imaging against serial section electron microscopy, and found similar contrast from metal staining, implying a similar level of biological interpretability between two modalities. Additional GABAergic neuronal nuclei labeling with peroxidase APEX2 (Lam *et al.*, 2014) provides cell type information. The multi-contrast imaging strategy they demonstrated (Kuan *et al.*, 2020) can guide the design of x-ray imaging experiments to maximize their structural information. Another approach is to use the natural distribution of metals in the brain to add functional information in unstained specimens, whether through atomic spectrometry (McAllum & Hare, 2019) or x-ray induced x-ray fluorescence (Bourassa & Miller, 2012), which can also be combined with the staining and labeling methods described above.

To image function-induced changes in dense neuroanatomy, one can rapidly freeze a specimen after sensory stimulus. One can then look for synaptic junctions that were

undergoing neurotransmitter release, as evidenced by fusing vesicles containing neurotransmitters captured "in action" at the release sites of the neuronal axon. This has been done decades ago using transmission electron microscopy, where electrical stimulation and slam freezing was followed either by freeze fracture (Heuser *et al.*, 1979), or freeze-substitution and uranyl acetate staining followed by serial sectioning (Heuser & Reese, 1981). A more modern approach (Watanabe *et al.*, 2013; Kavalali & Jorgensen, 2014; Watanabe *et al.*, 2014) has used cultured neurons infected with lentivirus expressing a rhodopsin derivative that can be triggered using blue light, followed by rapid high-pressure freezing, freeze-substitution, uranyl acetate staining, plastic embedding, and sectioning (Watanabe *et al.*, 2013). When combined with genetic targeting, in principle this type of approach could lead to a very sparse set of target synapses to search for using the adaptive scanning approaches discussed in section S3.

References

Aharon, M., Elad, M. & Bruckstein, A. (2006). *IEEE Transactions on Signal Processing*, **54**(11), 4311–4322.

Allahgholi, A., Becker, J., Delfs, A., Dinapoli, R., Göttlicher, P., Graafsma, H., Greiffenberg, D., Hirsemann, H., Jack, S., Klyuev, A., Krüger, H., Kuhn, M., Laurus, T., Marras, A., Mezza, D., Mozzanica, A., Poehlsen, J., Shalev, O. S., Sheviakov, I., Schmitt, B., Schwandt, J., Shi, X., Smoljanin, S., Trunk, U., Zhang, J. & Zimmer, M. (2019). *Nuclear Instruments and Methods in Physics Research A*, **942**, 162324.

Bourassa, M. W. & Miller, L. M. (2012). *Metallomics*, **4**(8), 721–738.

Bunk, O., Dierolf, M., Kynde, S., Johnson, I., Marti, O. & Pfeiffer, F. (2008). *Ultramicroscopy*, **108**(5), 481–487.

Candès, E. J., Romberg, J. K. & Tao, T. (2006). *Communications on Pure and Applied Mathematics*, **59**(8), 1207–1223.

Chang, C. & Sakdinawat, A. (2014). *Nature Communications*, **5**, 4243.

Clarke, N. I. & Royle, S. J. (2019). *Nature Communications*, **9**, 2604.

Cohn, D. A., Ghahramani, Z. & Jordan, M. I. (1996). *Journal of Artificial Intelligence Research*, **4**, 129–145.

Deng, J., Nashed, Y. S. G., Chen, S., Phillips, N. W., Peterka, T., Ross, R., Vogt, S., Jacobsen, C. & Vine, D. J. (2015). *Optics Express*, **23**(5), 5438–5451.

Deng, J., Preissner, C., Klug, J. A., Mashrafi, S., Roehrig, C., Jiang, Y., Yao, Y., Wojcik, M., Wyman, M. D., Vine, D., Yue, K., Chen, S., Mooney, T., Wang, M., Feng, Z., Jin, D., Cai, Z., Lai, B. & Vogt, S. (2019). *Review of Scientific Instruments*, **90**(8), 083701.

Depannemaecker, D., Santos, L. E. C., de Almeida, A.-C. G., Ferreira, G. B. S., Baraldi, G. L., Miqueles, E. X., de Carvalho, M., Costa, G. S. R., Marques, M. J. G., Scorza, C. A. & Rinkel, J. (2019). *ACS Chemical Neuroscience*, **10**, 3404–3408.

Dierolf, M., Thibault, P., Menzel, A., Kewish, C. M., Jefimovs, K., Schlichting, I., König, K. v., Bunk, O. & Pfeiffer, F. (2010). *New Journal of Physics*, **12**(3), 035017.

Donoho, D. L. (2006). *IEEE Transactions on Information Theory*, **52**(4), 1289–1306.

Dwivedi, P., Konijnenberg, S., Pereira, S. & Urbach, P. (2018). *Ultramicroscopy*, **192**(Phys. Rev. Lett. 92 2 2004), 29–36.

Edo, T. B., Batey, D. J., Maiden, A. M., Rau, C., Wagner, U., Pešić, Z. D., Waigh, T. A. & Rodenburg, J. M. (2013). *Physical Review A*, **87**(5), 053850.

Enders, B., Dierolf, M., Cloetens, P., Stockmar, M., Pfeiffer, F. & Thibault, P. (2014). *Applied Physics Letters*, **104**(17), 171104.

Fera, A., He, Q., Zhang, G. & Leapman, R. D. (2020). *Journal of Microscopy*, **277**(2), 71–78.

Genoud, C., Titze, B., Graff-Meyer, A. & Friedrich, R. W. (2018). *Frontiers in Neuroanatomy*, **12**, 76.

Guizar-Sicairos, M. & Fienup, J. R. (2008). *Optics Express*, **16**(10), 7264–7278.

Guizar-Sicairos, M., Johnson, I., Diaz, A., Holler, M., Karvinen, P., Stadler, H.-C., Dinapoli, R., Bunk, O. & Menzel, A. (2014). *Optics Express*, **22**(12), 14859–14870.

Gürsoy, D. (2017). *Optics Letters*, **42**(16), 3169–3172.

Hajmeer, M. N. & Basheer, I. A. (2003). *International Journal of Food Microbiology*, **82**(3), 233–243.

Henke, B. L., Gullikson, E. M. & Davis, J. C. (1993). *Atomic Data and Nuclear Data Tables*, **54**, 181–342.

Heuser, J. & Reese, T. (1981). *Journal of Cell Biology*, **88**(3), 564–580.

Heuser, J., Reese, T., Dennis, M., Jan, Y., Jan, L. & Evans, L. (1979). *Journal of Cell Biology*, **81**, 275–300.

Hieber, S. E., Bikis, C., Khimchenko, A., Schweighauser, G., Hench, J., Chicherova, N., Schulz, G. & Müller, B. (2016). *Scientific Reports*, **6**, 32156.

Howells, M., Beetz, T., Chapman, H., Cui, C., Holton, J., Jacobsen, C., Kirz, J., Lima, E., Marchesini, S., Miao, H., Sayre, D., Shapiro, D., Spence, J. & Starodub, D. (2009). *Journal of Electron Spectroscopy and Related Phenomena*, **170**, 4–12.

Hua, Y., Laserstein, P. & Helmstaedter, M. (2015). *Nature Communications*, **6**, 7923.

Huang, X., Lauer, K., Clark, J. N., Xu, W., Nazaretski, E., Harder, R., Robinson, I. K. & Chu, Y. S. (2015). *Scientific Reports*, **5**, 9074.

Huang, X., Yan, H., Ge, M., Öztürk, H., Nazaretski, E., Robinson, I. K. & Chu, Y. S. (2017). *Applied Physics Letters*, **111**(2), 023103.

Huang, X., Yan, H., Harder, R. J., Hwu, Y., Robinson, I. K. & Chu, Y. S. (2014). *Optics Express*, **22**(10), 12634–12644.

Jacobsen, C. (2020). *X-ray Microscopy.* Cambridge: Cambridge University Press.

Jacobsen, C., Deng, J. & Nashed, Y. S. G. (2017). *Journal of Synchrotron Radiation*, **24**(5), 1078–1081.

Jacobsen, C., Williams, S., Anderson, E., Browne, M. T., Buckley, C. J., Kern, D., Kirz, J., Rivers, M. & Zhang, X. (1991). *Optics Communications*, **86**, 351–364.

Jefimovs, K., Vila-Comamala, J., Pilvi, T., Rabbe, J., Ritala, M. & David, C. (2007). *Physical Review Letters*, **99**, 264801.

Ji, S., Xue, Y. & Carin, L. (2008). *IEEE Transactions on Signal Processing*, **56**(6), 2346–2356.

Jin, Q., Paunesku, T., Lai, B. P., Gleber, S.-C., Chen, S., Finney, L. A., Vine, D. J., Vogt, S., Woloschak, G. E. & Jacobsen, C. (2017). *Journal of Microscopy*, **265**(1), 81–93.

Jones, B., Jenkinson, I., Yang, Z. & Wang, J. (2010). *Reliability Engineering & System Safety*, **95**(3), 267–277.

Kaestner, A. P., Münch, B., Trtik, P. & Butler, L. (2011). *Optical Engineering*, **50**(12), 123201.

Kamaya, N., Homma, K., Yamakawa, Y., Hirayama, M., Kanno, R., Yonemura, M., Kamiyama, T., Kato, Y., Hama, S., Kawamoto, K. & Mitsui, A. (2011). *Nature Materials*, **10**(9), 682–686.

Kasthuri, N., Hayworth, K. J., Berger, D. R., Schalek, R. L., Conchello, J. A., Knowles-Barley, S., Lee, D., Vázquez-Reina, A., Kaynig, V., Jones, T. R., Roberts, M., Morgan, J. L., Tapia, J. C., Seung, H. S., Roncal, W. G., Vogelstein, J. T., Burns, R., Sussman, D. L., Priebe, C. E., Pfister, H. & Lichtman, J. W. (2015). *Cell*, **162**(3), 648–661.

Kavalali, E. T. & Jorgensen, E. M. (2014). *Nature Neuroscience*, **17**(1), 10–16.

Khimchenko, A., Bikis, C., Pacureanu, A., Hieber, S. E., Thalmann, P., Deyhle, H., Schweighauser, G., Hench, J., Frank, S., Müller-Gerbl, M., Schulz, G., Cloetens, P. & Müller, B. (2018). *Advanced Science*, **5**(6), 1700694.

Khimchenko, A., Deyhle, H., Schulz, G., Schweighauser, G., Hench, J., Chicherova, N., Bikis, C., Hieber, S. E. & Müller, B. (2016). *NeuroImage*, **139**(C), 26–36.

Kim, C., Kapfer, R. & Gruner, S. (2005). *Acta Crystallographica D*, **61**, 881–890.

Kirz, J. (1974). *Journal of the Optical Society of America*, **64**, 301–309.

Kreutz-Delgado, K., Murray, J. F., Rao, B. D., Engan, K., Lee, T.-W. & Sejnowski, T. J. (2003). *Neural Computation*, **15**(2), 349–396.

Kuan, A. T., Phelps, J. S., Thomas, L. A., Nguyen, T. M., Han, J., Chen, C.-L., Azevedo, A. W., Tuthill, J. C., Funke, J., Cloetens, P., Pacureanu, A. & Lee, W.-C. A. (2020). *Nature Neuroscience*, **23**(12), 1637–1643.

Lam, S. S., Martell, J. D., Kamer, K. J., Deerinck, T. J., Ellisman, M. H., Mootha, V. K. & Ting, A. Y. (2014). *Nature Methods*, **12**(1), 51–54.

Lewi, J., Butera, R. & Paninski, L. (2009). *Neural Computation*, **21**(3), 619–687.

Li, K., Ali, S., Wojcik, M. J., De Andrade, V., Huang, X., Yan, H., Chu, Y. S., Nazaretski, E., Pattammattel, A. & Jacobsen, C. (2020). *Optica*, **7**(5), 410–416.

Martin, T. & Koch, A. (2006). *Journal of Synchrotron Radiation*, **13**(2), 180–194.

Massimi, L., Bukreeva, I., Santamaria, G., Fratini, M., Corbelli, A., Brun, F., Fumagalli, S., Maugeri, L., Pacureanu, A., Cloetens, P., Pieroni, N., Fiordaliso, F., Forloni, G., Uccelli, A., de Rosbo, N. K., Balducci, C. & Cedola, A. (2019). *NeuroImage*, **184**, 490–495.

McAllum, E. J. & Hare, D. J. (2019). *Spectrochimica Acta Part B*, **156**, 20–32.

Mikula, S. & Denk, W. (2015). *Nature Methods*, **12**, 541–546.

Mohacsi, I., Vartiainen, I., Guizar-Sicairos, M., Karvinen, P., Guzenko, V. A., Müller, E., Färm, E., Ritala, M., Kewish, C. M., Somogyi, A. & David, C. (2015). *Optics Express*, **23**(2), 776.

Mohacsi, I., Vartiainen, I., Rösner, B., Guizar-Sicairos, M., Guzenko, V. A., McNulty, I., Winarski, R., Holt, M. V. & David, C. (2017). *Scientific Reports*, **7**, 43624.

Mokso, R., Cloetens, P., Maire, E., Ludwig, W. & Buffiere, J.-Y. (2007). *Applied Physics Letters*, **90**(14), 144104.

Moor, H. (1987). In *Cryotechniques in Biological Electron Microscopy*, edited by R. Steinbrecht & K. Zierold, pp. 175–191. Berlin: Springer-Verlag.

Munnig Schmidt, R. (2012). *Philosophical Transactions of the Royal Society of London A*, **370**(1973), 3950–3972.

O'Toole, E., Wray, G., Kremer, J. & McIntosh, J. R. (1993). *Journal of Structural Biology*, **110**, 55–66.

Pelz, P. M., Guizar-Sicairos, M., Thibault, P., Johnson, I., Holler, M. & Menzel, A. (2014). *Applied Physics Letters*, **105**, 251101.

Perrin, L., Carmona, A., Roudeau, S. & Ortega, R. (2015). *Journal of Analytical Atomic Spectrometry*, **30**(12), 2525–2532.

Ren, Y., Wang, Y., Zhou, G., He, Y., Xie, H., Du, G., Deng, B., Lin, X., Yang, G.-y. & Xiao, T. (2016). *Journal of X-ray Science and Technology*, **24**(1), 79–86.

Sang, X., Lupini, A. R., Unocic, R. R., Chi, M., Borisevich, A. Y., Kalinin, S. V., Endeve, E., Archibald, R. K. & Jesse, S. (2016). *Advanced Structural and Chemical Imaging*, **2**(1), 6.

Sayre, D., Kirz, J., Feder, R., Kim, D. M. & Spiller, E. (1977). *Science*, **196**, 1339–1340.

Schneider, G. (1997). *Applied Physics Letters*, **71**(16), 2242–2244.

Shahmoradian, S. H., Tsai, E. H. R., Diaz, A., Guizar-Sicairos, M., Raabe, J., Spycher, L., Britschgi, M., Ruf, A., Stahlberg, H. & Holler, M. (2017). *Scientific Reports*, **7**(1), 6291.

da Silva, J. C. & Menzel, A. (2015). *Optics Express*, **23**(26), 33812–33821.

da Silva, J. C., Pacureanu, A., Yang, Y., Bohic, S., Morawe, C., Barrett, R. & Cloetens, P. (2017). *Optica*, **4**(5), 492–495.

Stevens, A., Luzi, L., Yang, H., Kovarik, L., Mehdi, B. L., Liyu, A., Gehm, M. E. & Browning, N. D. (2018*a*). *Applied Physics Letters*, **112**(4), 043104.

Stevens, A., Yang, H., Hao, W., Jones, L., Ophus, C., Nellist, P. D. & Browning, N. D. (2018*b*). *Applied Physics Letters*, **113**(3), 033104.

Sullivan, S. Z., Muir, R. D., Newman, J. A., Carlsen, M. S., Sreehari, S., Doerge, C., Begue, N. J., Everly, R. M., Bouman, C. A. & Simpson, G. J. (2014). *Optics Express*, **22**(20), 24224–11.

Thibault, P. & Menzel, A. (2013). *Nature*, **494**(7435), 68–71.

Tong, S. (2001). *Active learning: theory and applications*. Ph.D. thesis, Department of Computer Science, Stanford University.

Töpperwien, M., Krenkel, M., Vincenz, D., Stöber, F., Oelschlegel, A. M., Goldschmidt, J. & Salditt, T. (2017). *Scientific Reports*, **7**, 42847.

Trampert, P., Bourghorbel, F., Potocek, P., Peemen, M., Schlinkmann, C., Dahmen, T. & Slusallek, P. (2018). *Ultramicroscopy*, **191**, 11–17.

Uhlén, F., Nilsson, D., Rahomäki, J., Belova, L., Schroer, C. G., Seiboth, F., Holmberg, A., Hertz, H. M. & Vogt, U. (2014). *Microelectronic Engineering*, **116**, 40–43.

Velazco, A., Nord, M., Béché, A. & Verbeeck, J. (2020). *Ultramicroscopy*, **215**, 113021.

Victor, T. W., O'Toole, K. H., Easthon, L. M., Ge, M., Smith, R. J., Huang, X., Yan, H., Chu, Y. S., Gürsoy, D., Ralle, M., Imperiali, B., Allen, K. N. & Miller, L. M. (2020). *Journal of the American Chemical Society*, **142**(5), 2145–2149.

Watanabe, S., Davis, M. W. & Jorgensen, E. M. (2014). In *Nanoscale Imaging of Synapses: New Concepts and Opportunities*, edited by U. V. Nägerl & A. Triller, pp. 43–57. New York, NY: Springer New York.

Watanabe, S., Rost, B. R., Camacho-Pérez, M., Davis, M. W., Söhl-Kielczynski, B., Rosenmund, C. & Jorgensen, E. M. (2013). *Nature*, **504**(7479), 242–247.

Wilke, V. (1983). *Proceedings SPIE*, **396**, 164–172.

Yao, Y., Deng, J., Klug, J. A., Jiang, Y., Wojcik, M. J., Nashed, Y. S. G., Preissner, C. A., Roehrig, C., Cai, Z., Cossairt, O., Vogt, S. & Lai, B. P. (2019). In *X-Ray Nanoimaging: Instruments and Methods IV*, edited by B. P. Lai & A. Somogyi, p. 111120G. SPIE.

Zhang, F., Peterson, I., Vila-Comamala, J., Diaz, A., Berenguer, F., Bean, R., Chen, B., Menzel, A., Robinson, I. K. & Rodenburg, J. M. (2013). *Optics Express*, **21**(11), 13592.

Zhang, Q., Lee, W.-C. A., Paul, D. L. & Ginty, D. D. (2019). *Nature Neuroscience*, **22**, 828–839.

Ziegler, D., Meyer, T. R., Amrein, A., Bertozzi, A. L. & Ashby, P. D. (2017). *IEEE/ASME Transactions on Mechatronics*, **22**(1), 381–391.
